# Innate cells and STAT1-dependent signals orchestrate vaccine-induced protection against invasive *Cryptococcus* infection

**DOI:** 10.1128/mbio.01944-24

**Published:** 2024-09-26

**Authors:** Keyi Wang, Vanessa Espinosa, Yina Wang, Alexander Lemenze, Yosuke Kumamoto, Chaoyang Xue, Amariliz Rivera

**Affiliations:** 1 Graduate School of Biomedical Sciences, New Jersey Medical School, Rutgers University, Newark, New Jersey, USA; 2 Department of Pediatrics and Center for Immunity and Inflammation, New Jersey Medical School, Rutgers University, Newark, New Jersey, USA; 3 Public Health Research Institute, New Jersey Medical School, Rutgers University, Newark, New Jersey, USA; 4 Department of Medicine and Center for Immunity and Inflammation, New Jersey Medical School, Rutgers University, Newark, New Jersey, USA; 5 Department of Microbiology, Biochemistry and Molecular Genetics, New Jersey Medical School, Rutgers University, Newark, New Jersey, USA; Albert Einstein College of Medicine, Bronx, New York, USA

**Keywords:** immunization, *Cryptococcus neoformans*, antifungal therapy, innate immunity, monocytes, neutrophils

## Abstract

Fungal pathogens are underappreciated causes of significant morbidity and mortality worldwide. In previous studies, we determined that a heat-killed, *Cryptococcus neoformans* fbp1-deficient strain (HK-fbp1) is a potent vaccine candidate. We determined that vaccination with HK-fbp1 confers protective immunity against lethal Cryptococcosis in an interferon γ (IFNγ)-dependent manner. In this study, we set out to uncover cellular sources and relevant targets of the protective effects of IFNγ in response to the HK-fbp1 vaccine. We found that early IFNγ production peaks at day 3 and that monocytes and neutrophils are important sources of this cytokine after vaccination. Neutralization of IFNγ at day 3 results in impaired CCR2^+^ monocyte recruitment and reduced differentiation into monocyte-derived dendritic cells (Mo-DC). In turn, depletion of CCR2^+^ cells prior to immunization results in impaired activation of IFNγ-producing CD4 and CD8 T cells. Thus, monocytes are important targets of innate IFNγ and help promote further IFNγ production by lymphocytes. We employed monocyte-fate mapper and conditional STAT1 knockout mice to uncover that STAT1 activation in CD11c^+^ cells, including alveolar macrophages, Mo-DCs, and monocyte-derived macrophages (Mo-Mac) is essential for HK-fbp1 vaccine-induced protection. Altogether, our aggregate findings suggest critical roles for innate cells as orchestrators of vaccine-induced protection against *Cryptococcus* infection.

**IMPORTANCE:**

The number of patients susceptible to invasive fungal infections across the world continues to rise at an alarming pace yet current antifungal drugs are often inadequate. Immune-based interventions and novel antifungal vaccines hold the promise of significantly improving patient outcomes. In previous studies, we identified a *Cryptococcus neoformans* mutant strain (Fbp1-deficient) as a potent, heat-inactivated vaccine candidate capable of inducing homologous and heterologous antifungal protection. In this study, we used a combination of methods together with a cohort of conditional knockout mouse strains to interrogate the roles of innate cells in the orchestration of vaccine-induced antifungal protection. We uncovered novel roles for neutrophils and monocytes as coordinators of a STAT1-dependent cascade of responses that mediate vaccine-induced protection against invasive cryptococcosis. This new knowledge will help guide the future development of much-needed antifungal vaccines.

## INTRODUCTION

Invasive fungal infections are estimated at an annual incidence of 6.5 million and 3.8 million deaths (1). With the increase of immunocompromised populations due to HIV infection, aging, and immunosuppressive treatments, the incidence of invasive fungal infection cases has risen significantly over the past several decades (2). Among them, cryptococcosis, caused by *Cryptococcus neoformans* (*Cn*) remains a difficult-to-treat infection that is a significant cause of mortality in susceptible patients worldwide (3). Protection against *C. neoformans* is dependent on T cells as demonstrated by the high susceptibility to cryptococcal meningitis in untreated AIDS patients with impaired function and/or low numbers of CD4^+^ T helper cells (4–6). It has been shown that in response to pulmonary fungal infection, naïve CD4^+^ T cells differentiate into diverse helper subsets that are distinguished by their cytokine profiles (7). Clinical studies have suggested that increased production of IFNγ, the hallmark cytokine of Th1 cells, correlates with a better prognosis of infected individuals (8–11). Conversely, Th2 responses, characterized by the secretion of IL-4, IL-5, and IL-13, are detrimental during cryptococcosis and exacerbate disease progression (12–15).

In contrast to the well-known function of T cells during *Cryptococcus* infection, the contributions of innate cells are not as well understood. Innate immune responses act as the first line of defense against multiple pathogens including fungi (16). Beyond physical barriers such as mucous membranes and epithelial cells, various immune cells such as monocytes, neutrophils, macrophages, and dendritic cells play important roles as first responders against infection (17, 18). Alveolar macrophages (AMs) are thought to play an integral role in anti-cryptococcal defense primarily by acting as key phagocytic cells (19). Studies in the literature report conflicting results on the role of CD11c^+^ macrophages in defense against *C. neoformans* (20). For example, some studies showed that depletion of AMs decreases the dissemination of *Cryptococcus*, and depletion of CD11c-expressing cells had increased neutrophil infiltration in the lung associated with severe lung inflammation (21). Other studies suggest that macrophages can serve as a “Trojan horse” to intracellular pathogens (22). In this case, *Cn* cells contained within host phagocytes are capable of egress from the lungs and dissemination to the CNS, where they accelerate disease progression (22). Similarly, monocytes have been reported to play beneficial or harmful roles to the host in response to *Cryptococcus* infection (23, 24). In chronic models of respiratory cryptococcosis, CCR2^+^ inflammatory monocytes have been shown to be beneficial to the host (25, 26). In that model, CCR2-deficient mice, develop a harmful Th2 response and have increased fungal burden and decreased numbers of lung macrophages (27). However, there is evidence of a detrimental impact of monocytes due to their role in mediating *Cryptococcus* dissemination and brain invasion (28). In addition to the controversial role of monocytes, the role of neutrophils in *Cryptococcus* infection is also poorly defined (29). Studies have reported protective roles for neutrophils that include serving as a source of IL-17A and as necessary effectors for clearing fungal cells in the brain and the lung (30, 31). However, other studies demonstrated that the depletion of monocytes and neutrophils together improved mouse survival and reduced overall lung damage, suggesting a harmful role for neutrophils (32).

In aggregate, previous studies indicate that the interaction between *Cryptococcus* and the host is a complicated interplay that ultimately dictates two widely divergent outcomes: (i) a protective Th1 response is provoked, infection is resolved, or infection persists but is controlled in a dormant state to co-exist with the host; (ii) a harmful Th2 response is induced, the local infection spreads to the brain and lethal cryptococcal meningitis ensues. To date, there is no commercially available vaccine specifically designed for the prevention of *Cryptococcus* infection in humans but there is a clear medical need for such a vaccine to help protect susceptible populations (33). Encouragingly, proof-of-principle studies in mice have illustrated the feasibility of inducing vaccine-mediated protection against *Cryptococcus*. Thus, a detailed understanding of the protective mechanisms of vaccine-induced protection in animal models can help guide the design of protective antifungal vaccines for humans. Various anti-*Cryptococcus* vaccines have been explored and tested in animal models (34–38). Protective live-attenuated approaches include a sterylglucosidase mutant strain (*Sgl1*Δ), a chitosan-deficient strain (cda1Δ*2*Δ*3*Δ), a mutant overexpressing the transcription factor Znf2 (*Znf2^OE^*) (that controls yeast-to-hypha differentiation), and a transgenic protective cytokine IFNγ-producing strain (H99γ) (37, 39–41). The use of attenuated *Cryptococcus* strains as vaccines offers several advantages, including lower production costs and the capacity to trigger robust and long-lasting host responses (38). Importantly, some of these experimental strategies have employed killed preparations as a substitute for live organisms, providing a safer approach for vaccination of hosts with various underlying immune dysfunctions that render them susceptible to fungal infections. In previous studies, we identified the F-box protein 1 (Fbp1) as a novel virulence factor in *C. neoformans* strain H99. Fbp1 functions as a subunit of the SCF^Fbp1^ E3 ligase complex, a key component of the ubiquitin-mediated proteolytic pathway that targets specific proteins for ubiquitination and subsequent degradation (42, 43). We found that the *fbp1*Δ mutant strain can elicit superior protective Th1 host immunity, and a heat-killed fbp1 deficient strain (HK-fbp1) is a potent vaccine candidate that induces protection against infection with the virulent parental strain *Cn*-H99 in murine models (34, 44). We found that mice defective in IFNγ responsiveness (IFNγR^−/−^ mice) were not protected after HK-fbp1 vaccination and were as susceptible to infection as unvaccinated mice (44). HK-fbp1 immunization was unable to protect mice from *Cn*-H99 infection in mice depleted of both CD4^+^ and CD8^+^ T cells, but mice with either CD4^+^ or CD8 T^+^ cells were fully protected. Therefore, HK-fbp1 vaccine-induced protection is dependent on T cells and IFNγ signaling (44).

In this study, we set out to define the underlying mechanisms that contribute to the *in vivo* response elicited by HK-fbp1 vaccination. We hypothesized that innate cells are critical in the activation of IFNγ-dependent defense. We found that monocytes and neutrophils are important sources of early production of IFNγ and that depletion of CCR2^+^ cells during priming affects protective Th1 T cell activation and differentiation. We tracked the fate of monocyte-derived cells in the lung after Hk-fbp1 vaccination and determined that monocytes gave rise to monocyte-derived alveolar macrophages that showed a gene expression signature dominated by IFN-stimulated genes. Tissue-derived AMs also showed a dominant IFN signature thus suggesting that various innate cell subsets are targets of IFNs in this vaccine model. We further identified that CD11c^+^ cells, including AMs, Mo-DCs, and Mo-Mac are key mediators of vaccine-induced protection in a STAT1-dependent manner. Overall, our study uncovered detailed contributions of innate cells to vaccine-induced protection by serving as key sources of innate IFNγ, helping in the activation of T cells after immunization, and as mediators of vaccine-induced protection against lethal *Cn*-H99 infection.

## RESULTS

### IFNγ is critical for the activation of innate and adaptive responses upon vaccination with HK-fbp1

**Fig 1:**
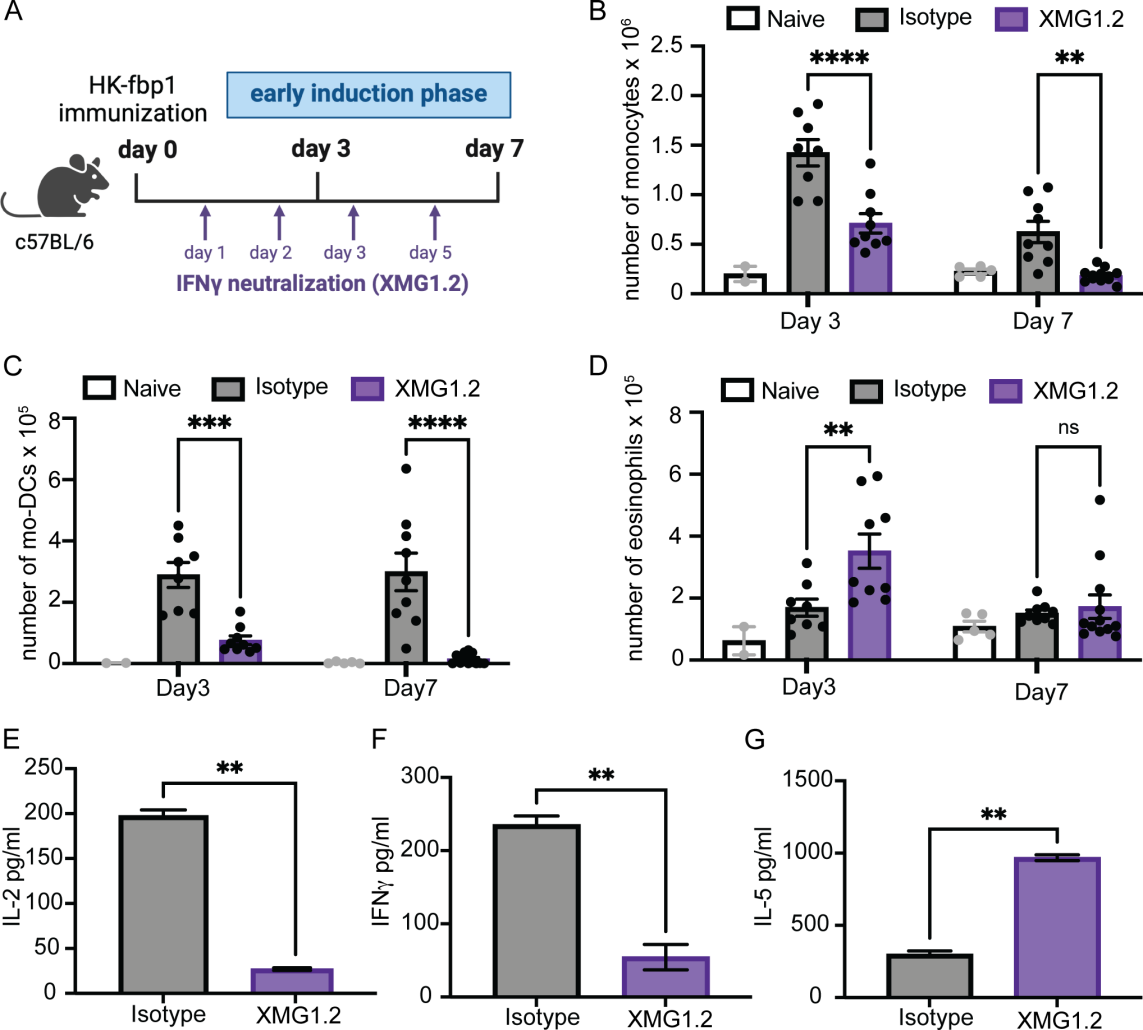
IFNγ is critical for the activation of innate and adaptive responses to vaccination with HK-fbp1. (**A**) Graphic summary of the experimental approach. C57BL/6 (WT B6) mice were vaccinated with HK-fbp1 on day 0 and sacrificed on day 3 and day 7 post-vaccination. Mice received 200 μg of XMG1.2 or isotype control antibody for neutralization of IFNγ on days +1, +2, +3, and +5. (B to D) Immune cell populations in the lung were identified by flow cytometry as detailed in Material and Methods. (**B**) The total number of monocytes, Mo-DCs (**C**), and eosinophils (**D**) present in the lung at day 3 and day 7 post-vaccination. Each symbol represents one mouse. (**E–G**) CD4^+^ T cells were purified from lung-draining lymph nodes and stimulated with *Cryptococcus* antigens for 72 hours. Levels of IL-2, IFNγ, and IL-5 secreted after stimulation were measured in culture supernatant by ELISA. The data shown are cumulative from two independent experiments with at least five mice per group and are depicted as the mean values ± standard errors of the means. ns, not significant; **, *P* < 0.01; ***, *P* < 0.001; ****, *P* < 0.0001 (B to D, determined by two-way analysis of variance [ANOVA] nonparametric test for multiple comparisons; E to G, determined by nonparametric *t*-test using Prism software).

In order to test the potential role of IFNγ during the early induction phase after vaccination we neutralized IFNγ during the first week of vaccination and examined the impact on innate cell recruitment and T cell activation. To this end, C57BL/6 (WTB6) mice were vaccinated with HK-fbp1 on day 0 and sacrificed on day 3 and day 7 post-vaccination. Mice received anti-IFNγ antibody (Clone XMG1.2) on days +1, +2, +3, and +5 to neutralize the effect of IFNγ during the initial vaccine-induced host response (Fig. 1A). The dose and timing of XMG1.2 used in this study is accordance with previous work that demonstrated the effective neutralization of IFNγ *in vivo* and subsequent susceptibility to *Toxoplasma gondii* infection that was comparable to IFNγ^−/−^ mice (45). We found that neutralization of early production of IFNγ resulted in reduced monocyte recruitment (Fig. 1B) and impaired monocyte maturation into Mo-DCs (Fig. 1C). This impact started at day 3 post-vaccination and continued to day 7 post-vaccination. Neutralization of early production of IFNγ resulted in increased eosinophil (linked to type 2 immune responses) recruitment at day 3 (Fig. 1D). Our previous studies showed that vaccination with HK-fbp1 could not protect lymphocyte-deficient mice (RAG^−/−^ mice) from a challenge with virulent H99, suggesting that vaccination-induced protection is dependent on adaptive immunity (34). Moreover, we previously determined that CD4^+^ or CD8^+^ T cells are sufficient for vaccine-induced protection, but depletion of both T cell subsets is detrimental (44). Thus, throughout this study, we monitor the activation of CD4^+^ and CD8^+^ T cells after vaccination. We observed that IFNγ neutralization resulted in decreased recruitment of CD4^+^ and CD8^+^ T cells to the lung and airways (Fig. S1A through C) thus suggesting that innate IFNγ is needed for the activation of T cell responses. We also examined whether IFNγ neutralization affected antigen-specific CD4^+^ T cell responses. To test this, we checked T cell recall responses of CD4^+^ T cells isolated from the lung-draining mediastinal lymph nodes (mLNs) of vaccinated mice using previously developed methods (34, 44). Cytokine secretion in the presence of sonicated *Cryptococcus* antigens was examined by enzyme-linked immunosorbent assay (ELISA) after 72 hours of culture. CD4^+^ T cells recovered from IFNγ-neutralized mice showed minimal production of IL-2 (Fig. 1E) and significantly reduced secretion of protective IFNγ (Fig. 1F) and IL17 A (Fig. S1D) as compared to T cells isolated from control, HK-fbp1 vaccinated mice. In contrast, levels of IL-5 production by isolated CD4^+^ T cells were relatively increased in the same culture conditions (Fig. 1G). Therefore, our data suggest that early sources of IFNγ are key for the proper activation of Mo-DCs and optimal priming and differentiation of Th1 CD4^+^ T cells during the initial induction phase after HK-fbp1 vaccination.

### Monocytes and neutrophils are important sources of IFNγ during the early induction phase after HK-fbp1 vaccination

**Fig 2:**
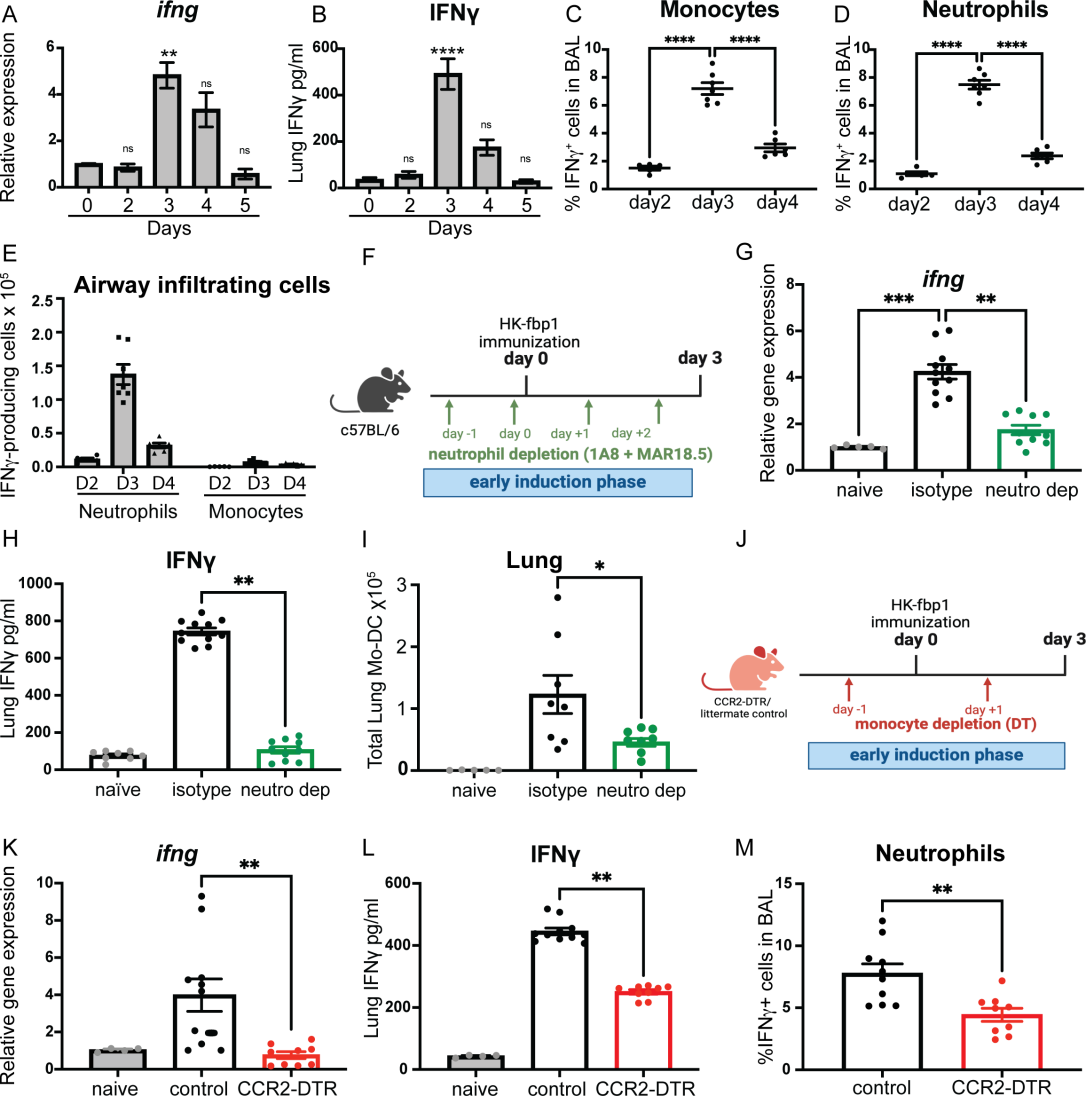
Monocytes and neutrophils are important sources of IFNγ during the early induction phase of HK-fbp1 vaccination. (**A and B**) WT B6 mice were vaccinated with HK-fbp1 5 × 10^7^ at day 0, and sacrificed on days 2, 3, 4, and 5 after vaccination. Kinetics of IFNγ RNA expression (**A**) and protein expression (**B**) in the lung of mice were analyzed at different time points after HK-fbp1 vaccination. Data shown are mean ± SEM of five mice per time point. (**C and D**) The frequency of IFNγ-producing innate cells after HK-fbp1 vaccination was examined by intracellular cytokine staining (ICCS). (**E**) The total number of IFNγ-producing innate cells recovered from BALF of mice vaccinated with HK-fbp1 2, 3, and 4 days prior to analysis. (**F**) Graphic summary of the experimental approach. WTB6 mice were vaccinated with HK-fbp1 on day 0 and sacrificed on day 3 post-vaccination. WT B6 mice were itraperitoneally (i.p.) injected with 100μg 1A8 antibody on day −1 and day +1. On day 0, mice were vaccinated with HK-fbp1. On day 0 and day +2, mice were i.p. injected with 100μg of MAR18.5 antibody. Control antibody 2A3 100μg was i.p. injected into mice on day −1 and day +1. (**G and H**) IFNγ RNA (**G**) and protein expression (**H**) were examined in the lungs of mice on day 3 after HK-fbp1 vaccination. (**G**) IFNγ RNA expression was determined by quantitative reverse transcription polymerase chain reaction (qRT-PCR) using TaqMan probes and normalized to GAPDH. (**H**) IFNγ protein level in lung homogenates was measured by ELISA. Data shown are mean ± SEM of five mice per time point. (**I**) Total number of Mo-DC in the lung as determined by flow cytometry. Each symbol represents one mouse. (**J**) Graphical summary of the experimental approach. CCR2-DTR mice and control CCR2-DTR negative littermates received 250 ng of diphtheria toxin i.p. on day −1 to vaccination and day +1 after vaccination to maintain depletion. (**K and L**) IFNγ RNA expression (**K**) and protein expression (**L**) in the lung of mice at day 3 after HK-fbp1 vaccination. All RNA gene expression was determined by qRT-PCR using TaqMan probes and normalized to the GAPDH housekeeping gene. Protein level was measured by ELISA. (**M**) Percent of IFN-γ-producing neutrophils recovered from the airways of control and CCR2-DTR mice as determined by ICCS. Each symbol represents one mouse. *, *P* < 0.05; **, *P* < 0.01; ***, *P* < 0.001; ****, *P* < 0.0001 (determined by one-way ANOVA nonparametric test for multiple comparisons using Prism software).

In order to determine the source of IFNγ after HK-fbp1 vaccination, we performed a kinetics analysis of IFNγ expression during the first 5 days after immunization. We found that IFNγ was up-regulated in the lung after HK-fbp1 vaccination and that RNA and protein levels were steadily increased with a peak response at day 3 post-vaccination (Fig. 2A and B). We also analyzed IFNγ-producing cells in the airways (bronchoalveolar lavage fluid-BALF) and found that neutrophils and monocytes were able to produce IFNγ during the early induction phase after vaccination (Fig. 2C and D; Fig. S2A). IFN-γ-production was also observed in NK cells and alveolar macrophages (data not shown). These results identified the presence of IFNγ-producing innate cells in the airway after mice received HK-fbp1 vaccination. Furthermore, the peak of IFNγ production overlapped with the kinetics of innate cell-derived IFNγ in the airway. Among these cell populations, neutrophils were the most abundant cells in the airway after vaccination (Fig. 2E). Therefore, we hypothesized that neutrophils are a significant cellular source of early IFNγ expression. In order to test the contribution of IFNγ-producing neutrophils during the early induction phase of HK-fbp1 vaccination, we removed neutrophils by antibody-mediated depletion with anti-Ly6G (Clone 1A8) combined with a secondary anti-rat IgG2aκ light chain (Clone MAR18.5) (Fig. 2F). This combined antibody treatment resulted in highly efficient depletion of neutrophils *in vivo* (46). Depletion of neutrophils for 3 days resulted in diminished IFNγ expression as measured by RNA and protein levels in the lung (Fig. 2G and H). These results suggest that neutrophils are a relevant source of IFNγ at day 3 post-HK-fbp1 vaccination. Previous studies showed that the depletion of neutrophils could affect the recruitment and activation of CCR2^+^ inflammatory monocytes *in vivo* (47). Consistently, we found a decreased accumulation of Mo-DC in the lung of neutrophil-depleted mice (Fig. 2I). Since we observed that Mo-DCs were reduced in IFNγ-neutralized mice (Fig. 1C), we hypothesize that neutrophil depletion affects total levels of IFNγ (Fig. 2G and H) and consequently results in reduced Mo-DCs (Fig. 2I).

In addition to neutrophils, we observed that monocytes are also producers of IFNγ. To test the possible impact of CCR2^+^ monocytes on overall IFNγ production, we depleted monocytes using CCR2 depleter mice (CCR2-DTR), which express a functional diphtheria toxin receptor (DTR) under the control of the CCR2 promoter. Mice were injected with diphtheria toxin (DT) 1 day before and 1 day after immunization with the HK-fbp1 vaccine to maintain depletion (Fig. 2J). Animals were sacrificed on day 3 post-vaccination to examine the expression of IFNγ in the lung. We found that IFNγ RNA level is diminished in the lungs of CCR2-DTR mice, while expression in their littermate controls was not affected (Fig. 2K). The lung IFNγ protein level was also reduced in CCR2-DTR mice, indicating that CCR2^+^ monocytes are also a source of IFNγ during the early induction phase after HK-fbp1 vaccination (Fig. 2L). Moreover, we found that the depletion of CCR2^+^ cells in mice did not affect neutrophil recruitment (Fig. S2B). Instead, monocyte depletion affected the capacity of neutrophils to produce IFNγ (Fig. 2M), suggesting that the cellular interplay between monocytes and neutrophils during HK-fbp1 vaccination results in optimal IFNγ production. In aggregate, these results suggest that monocytes and neutrophils serve as important sources of early IFNγ during the induction phase of HK-fbp1 vaccination.

### CCR2^+^ monocytes are required for CD4^+^ T cell differentiation and optimal entry to the lung and airways

**Fig 3:**
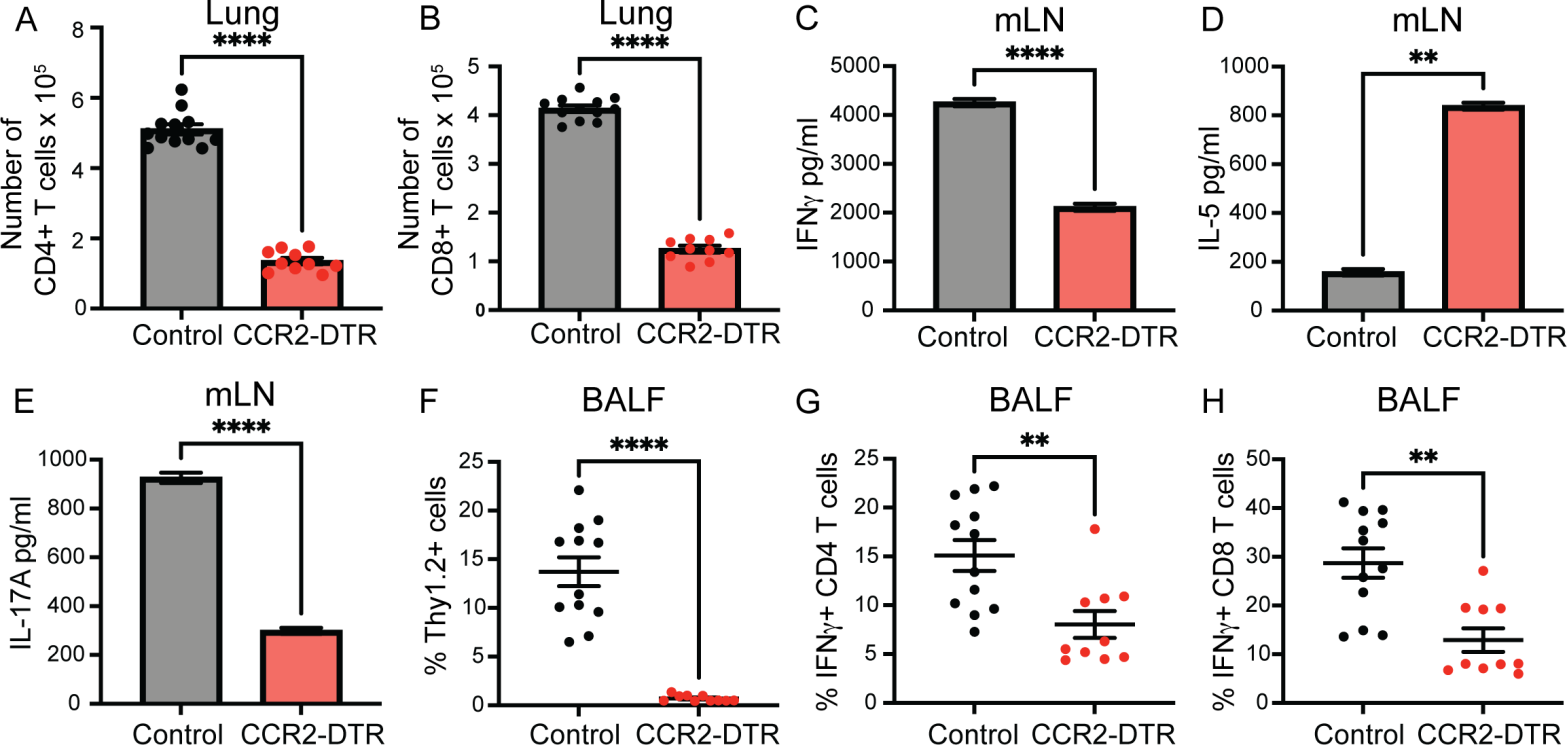
CCR2^+^ monocytes are required for CD4^+^ T cell differentiation and optimal entry to the airway. CCR2-DTR mice and control CCR2-DTR negative littermates mice were vaccinated with HK-fbp1 5 × 10^7^ on day 0 and sacrificed on day 7 post-vaccination. All mice received 250 ng of diphtheria toxin i.p. 1 day before vaccination and every other day post-vaccination to maintain depletion. (**A and B**) CD4^+^ and CD8^+^ T cell recruitment to the lungs of CCR2-depleted mice and control littermates was examined by flow cytometry. Each symbol represents one mouse. (**C–E**) CD4^+^ T cells were isolated from the lung-draining lymph node and re-stimulated with *Cryptococcus* antigens *ex vivo* for 72 hours. Secretion of IFNγ, IL-5, and IL-17A levels was examined in culture supernatant by ELISA. The data shown are mean ± SEM. (**F**) Percentage of total Thy1.2^+^ T cells recovered from BALF as analyzed by flow cytometry. Each symbol represents one mouse. (**G and H**) Cytokine expression was analyzed by ICCS and measured by flow cytometry. The frequencies of IFNγ-producing CD4^+^ T cells (**G**) and IFNγ-producing CD8^+^ T cells (**H**) in BALF were analyzed. Each symbol represents one mouse. Data are cumulative for three independent experiments with four mice per group. **, *P* < 0.01; ****, *P* < 0.0001 (determined by nonparametric *t*-test for comparison).

HK-fbp1-induced protection against homologous H99 infection depends on the proper activation and differentiation of Th1 and Th17 responses in order for optimal vaccine-induced protection to be elicited (34, 44). In previous studies, we determined that CCR2^+^ monocytes are required to facilitate the activation of CD4^+^ T cell responses during pulmonary fungal infection (34). We thus examined the impact of CCR2^+^ monocyte removal on CD4^+^ T cell activation at day 7 after immunization. We depleted monocytes by DT injection in CCR2-DTR mice 1 day before and every other day to maintain depletion for 7 days post-HK-fbp1 vaccination. On day 7, we sacrificed mice and examined CD4^+^ and CD8^+^ T cell recruitment and their cytokine profile in cells recovered from the BALF. We found that depletion of CCR2^+^ monocytes results in diminished recruitment of both CD4^+^ and CD8^+^ T cells to the lung of HK-fbp1-vaccinated mice (Fig. 3A and B). In contrast, depletion of CCR2^+^ monocytes did not affect neutrophil numbers in the lung at day 7 post-vaccination (Fig. S3A). Based on previous studies, we hypothesized that the reduced appearance of T cells in the airways of CCR2^+^ monocyte-depleted mice could be due to impaired priming of fungus-specific T cells in the mLNs (48). Therefore, we tested *Cn*-specific CD4^+^ T cell responses and found that CD4^+^ T cells recovered from the mLN of vaccinated littermate controls rapidly produced protective IFNγ and IL-17A (Fig. 3C and E). In contrast, CD4^+^ T cells recovered from vaccinated CCR2-depleted mice produced limited amounts of IFNγ and IL-17A, but they produced increased amounts of IL-5 (Fig. 3C to E). We found a significant decrease in total T cells recovered from the BALF in CCR2-DTR mice (Fig. 3F). Moreover, the few cells recovered showed significant reductions of IFNγ-producing CD4^+^ T and IFNγ-producing CD8^+^ T cells in CCR2-DTR mice as compared to their littermate controls (Fig. 3G and H), while IL-13-producing CD4^+^ T cells remain the same (Fig. S3B). In aggregate, these results suggest that the removal of CCR2^+^ inflammatory monocytes affects the proper activation of Th1 and Th17 responses while also allowing the differentiation of harmful Th2 cells. Together with our previous observations, our findings suggest that CCR2^+^ monocytes are needed for the proper production of innate IFNγ directly and by promoting IFNγ by neutrophils. The limited availability of IFNγ is likely linked to the observed defects in Th1 CD4^+^ T cell differentiation. Furthermore, our data indicate that CCR2^+^ monocytes are required for the proper activation and differentiation of Th1 CD4^+^ T cells. This observation is consistent with previous studies that demonstrated a direct role for CCR2^+^ monocytes in antigen transport to the mLN in the context of pulmonary fungal infection (48).

### CCR2^+^ monocytes and CD11c^+^ cells are critical for protective Th1 differentiation during late effector phase

**Fig 4:**
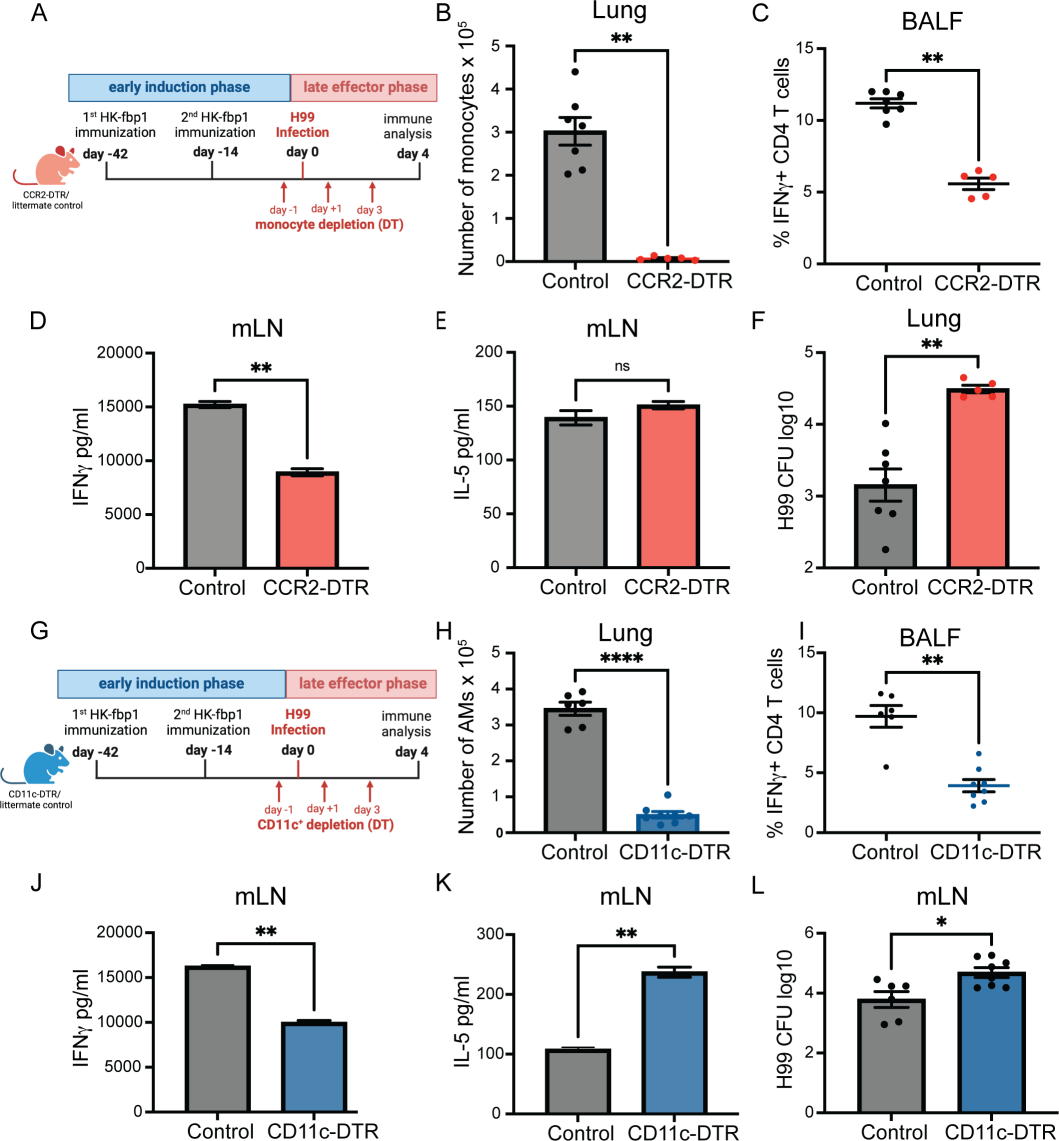
CCR2^+^ monocytes and CD11c^+^ cells are critical for Th1 differentiation during the late effector phase. (**A**) Graphical summary of the experimental approach. CCR2-DTR mice and control CCR2-DTR negative littermates were vaccinated with 5 × 10^7^ HK-fbp1 on day −42 and boosted with the same dose of vaccine on day −14. On day 0, mice were infected with live 10^4^ H99. All mice received 250 ng of diphtheria toxin i.p. on days −1, +1, +3, and sacrificed on day 4 post-H99 infection. (**B**) The total number of CCR2^+^ monocytes in the lung was examined by flow cytometry. Each symbol represents one mouse. (**C**) Percentage of IFNγ-producing CD4^+^ T cells in the airways as measured by ICCS and flow cytometry. Each symbol represents one mouse. (**D and E**) CD4^+^ T cells were isolated from the lung-draining lymph node and re-stimulated with *Cryptococcus* antigens *ex vivo* for 72 hours. Secretion of IFNγ and IL-5 levels were examined in culture supernatant by ELISA. The data shown are mean ± SEM. (**F**). H99 fungal burden in the lungs of vaccinated mice infected with 10^4^ H99 at day 4 (post-infection (p.i.). (**G**) Graphical summary of the experimental approach. CD11c-DTR and their WT littermate control mice were vaccinated with HK-fbp1 on day −42 and boosted with the same dose of vaccine on day −14. On day 0, mice were infected with live 10^4^ H99. All mice received 250 ng of diphtheria toxin i.p. on day −1, +1, +3, and +5, and sacrificed on day 7 post-H99 infection. (**H**) The total number of Live, CD45^+^ SiglecF^+^ CD11c^+^ cells in the lung was examined by flow cytometry. Each symbol represents one mouse. (**I**) The percentage of IFNγ-producing CD4^+^ T cells recovered from airways was analyzed by ICCS and measured by flow cytometry. Each symbol represents one mouse. (**J and K**) CD4^+^ T cells were isolated from the lung-draining lymph node and re-stimulated with *Cryptococcus* antigens *ex vivo* for 72 hours. Secretion of IFNγ and IL-5 levels were examined in culture supernatant by ELISA. The data shown are mean ± SEM. (**L**) Fungal burdens in the lungs of vaccinated mice infected with 10^4^ H99 were examined at day 7 p.i. Each symbol represents one mouse. Data are cumulative from two independent experiments with five mice per group. ns, not significant; *, *P* < 0.05; **, *P* < 0.01; ***, *P* < 0.001; ****, *P* < 0.0001 (as determined by nonparametric *t*-test for comparison with Prism software).

Our findings thus far indicate that CCR2^+^ monocytes are critical for optimal IFNγ production during the early induction phase of protective responses after HK-fbp1 vaccination. We hypothesize that CCR2^+^ monocytes not only serve as a critical cytokine-producer cell, but that their derivative cells, Mo-Mac and Mo-DC (both CD11c^+^ cells) could also be important for T cell re-expansion in vaccinated mice after H99 infection. In order to test the potential role of CCR2^+^ monocytes and their derivative cells (CD11c^+^) cells as effectors of vaccine-induced protection, we employed CCR2-DTR mice and CD11c-DTR mice to selectively remove CCR2^+^ cells or CD11c^+^ cells, respectively, during the late effector phase. We vaccinated CCR2-DTR or CD11c-DTR mice along with their littermate control mice with HK-fbp1 on day −42 and a boost vaccine on day −14. On day 0, these mice were infected with live H99. During the effector phase, we injected DT in mice to deplete all cells that expressed CCR2^+^ (Fig. 4A) or CD11c^+^ (Fig. 4G). Mice were sacrificed on day 4 or day 7 post-H99 infection for comparison of immune analyses and pulmonary fungal burdens. We confirmed that the total number of CCR2^+^ monocytes or CD11c^+^ cells was significantly reduced in CCR2-DTR or CD11c-DTR mice compared to their vaccinated littermate controls, respectively (Fig. 4B and H). We determined the impact of the depletion of CCR2^+^ or CD11c^+^ cells in the late effector phase by examining the balance of Th1 and Th2 responses in BALF and lymph nodes. We found that the removal of CCR2^+^ or CD11c^+^ innate cells resulted in reduced infiltration of IFNγ- (Fig. S4A) and IL-17-producing CD4^+^ T cells (Fig. S4D) in airways. We also observed decreased secretion of IFNγ (Fig. 4D and J) and IL-17 (Fig. S4B and E) by CD4^+^ T cells purified from the mLN of CCR2^+^ or CD11c^+^ depleted mice as compared to littermate controls. In contrast, secretion of IL-5 was unchanged (Fig. 4E) or increased (Fig. 4K) in the same cultures. Meanwhile, IL-2 secretion by re-stimulated CD4^+^ T cell was increased in mice depleted of CCR2^+^ (Fig. S4C) or CD11c^+^ (Fig. S4F) cells compared to their vaccinated littermate controls, suggesting preserved initial activation of CD4^+^ T cells. Furthermore, we found that the removal of CCR2^+^ monocytes or CD11c^+^ innate cells significantly increased lung fungal burden at day 4 post-infection with *Cn*-H99 (Fig. 4F and L). Based on our aggregate observations, we conclude that CCR2^+^ monocytes and CD11c^+^ cells are required during the late effector response of vaccine-induced immunity likely due to a combination of effector mechanisms that may include a role as direct effectors of fungal cell control as well as via regulation of optimal T cell responses.

### CD11c^+^ cells acquire a gene signature of IFN-inducible genes in response to vaccination with HK-fbp1

**Fig 5:**
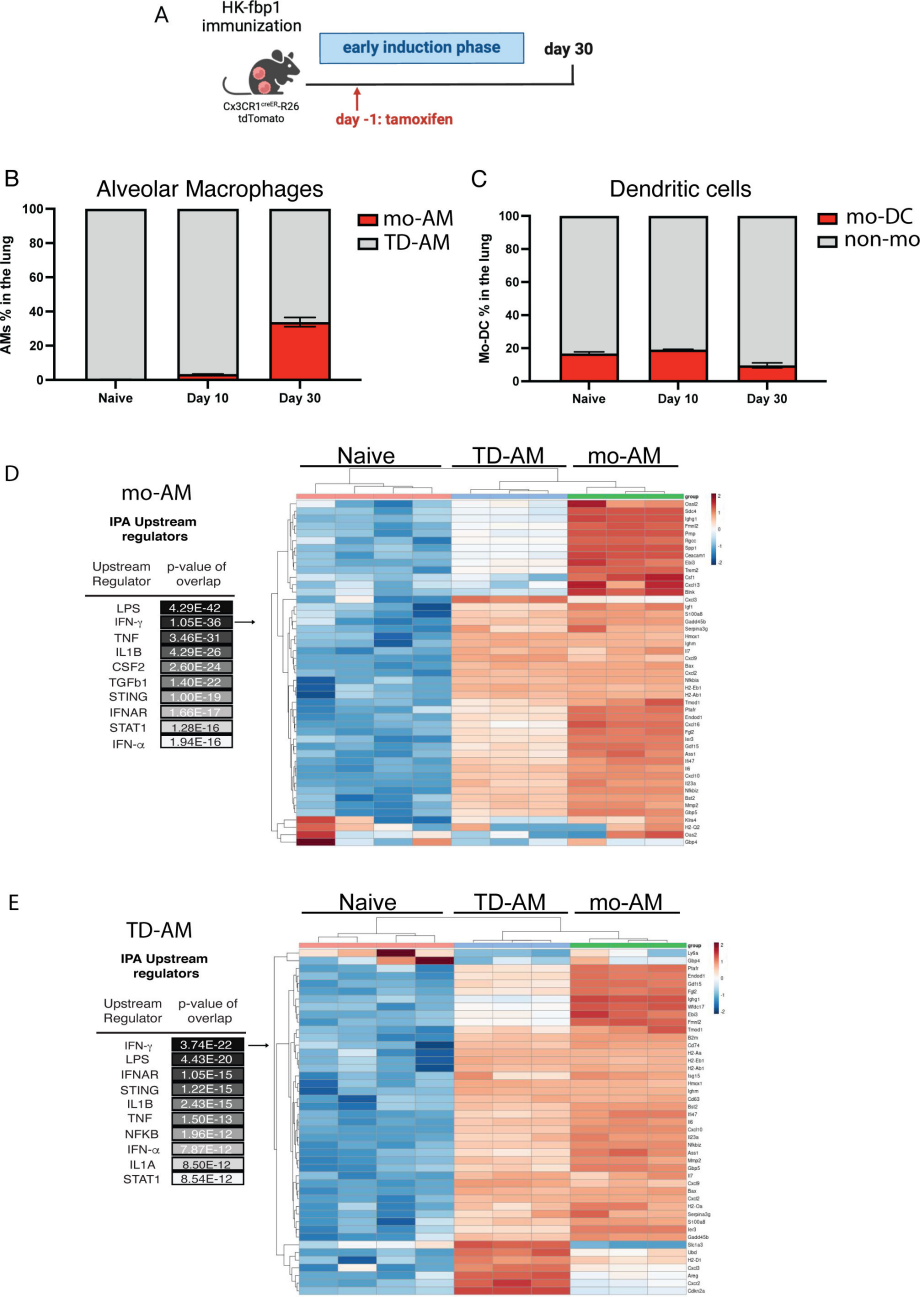
Monocyte-derived and tissue-derived AMs show evidence of sustained IFNγ gene signature. (**A**) CX3CR1^creER^ × R26TdTomato^fl/fl^ mice were vaccinated with HK-fbp1 and sacrificed on day 30 post-vaccination. (**B**). Percentage of AMs (CD45^+^ DAPI^−^ CD11c^+^ SiglecF^hi^) identified as TD-AM (TdT^-^) or mo-AM (TdT^+^) in the lung at various times after vaccination. (**C**) Percentage of dendritic cells (CD45^+^ DAPI^-^ CD11c^+^ SiglecF^lo^) identified as mo-DCs (TdT^+^) or non-mo-DC (TdT^-^). (**D**) Ingenuity Pathway analysis for upstream regulators in differentially expressed genes by Mo-AM vs Naïve control. Heat map of differentially expressed, IFNγ-regulated genes between naïve AM, Mo-AM and TD-AM from HK-fbp1 vaccinated mice ranked by log2 fold change.(**E**). Ingenuity pathway analysis for upstream regulators in differentially expressed genes by TD-AM vs naïve control. Heat map of differentially expressed, IFNγ-regulated genes between naïve AM, Mo-AM, and TD-AM from HK-fbp1 vaccinated mice ranked by log2 fold change.

Our aggregate data thus far indicate that CCR2^+^ monocytes are important producers and targets of IFNγ during the initial activation of protective T cells after HK-fbp1. Our data further suggest that CCR2^+^ and CD11c^+^ cells are also important during the effector phase. The relationship between CCR2^+^ monocytes and CD11c^+^ cells is complex where monocytes can be precursors of various CD11c^+^ populations including macrophage and dendritic cell subsets. In the lung, monocytes have been reported to replenish alveolar macrophages after infection (49, 50). Alveolar macrophages are considered key effectors of anti-cryptococcal control and were effectively depleted in both CCR2-DTR and CD11c-DTR mice (data not shown). Based on our observations, we hypothesize that the important roles observed for both CCR2^+^ and CD11c^+^ cells might be connected to the interrelationship of these populations where CCR2^+^ monocytes give rise to important CD11c^+^ effectors that no longer express CCR2. Thus, we set out to examine whether monocytes give rise to alveolar macrophages after HK-fbp1 immunization. To this end, we employed two models of monocyte-fate mapping: CCR2^CreER^ and CX3CR1^CreER^ inducible cre mice crossed to Rosa26^floxedTdTomato^ mice (Fig. 5A). A representative flow plot of gating is shown in Fig. S5A. We found that both models showed increased accumulation of monocyte-derived alveolar macrophages (Mo-AM) in the lungs of HK-fbp1 immunized mice (Fig. 5B and data not shown). Consistent with our previous study, we observed the increased accumulation of TdTomato positive, monocyte-derived AMs (Mo-AM), in the lung of HK-fbp1 immunized mice over time (49). In contrast, the number of Mo-DCs diminished over time (Fig. 5C). We then employed the fate mapper model to sort Mo-AM and tissue-derived AMs (TD-AM, non-red) from the lung of HK-Fbp1 immunized mice and examined their transcriptional response by RNAseq. We were particularly interested in examining whether these populations had evidence of IFN responsiveness and whether they would be equally or differentially impacted by HK-fbp1 immunization as compared to AMs isolated from unchallenged control mice. Principal component analysis indicated that each AM population had distinct transcriptional responses (Fig. S5B). Overall gene expression patterns visualized by volcano plots (Fig. S5C and D) suggested that Mo-AM and TD-AM maintained differential gene expression as compared with naïve cells with Mo-AM showing more robust transcription. Ingenuity pathway analysis for upstream activators of gene expression signatures seen in Mo-AM and TD-AM predicted IFNγ as a key regulator of both populations (Fig. 5D and E). Heat maps for these IPA-identified genes are shown in Fig. 5D and E. In aggregate, these analyses suggest that monocyte-derived and tissue-derived AMs are activated by HK-fbp1 vaccination and both show evidence of responsiveness to IFNγ which is a key mediator of vaccine-induced protection in this model.

### STAT1 expression on CD11c^+^ cells is required for maintaining a protective Th1 response after vaccination

**Fig 6:**
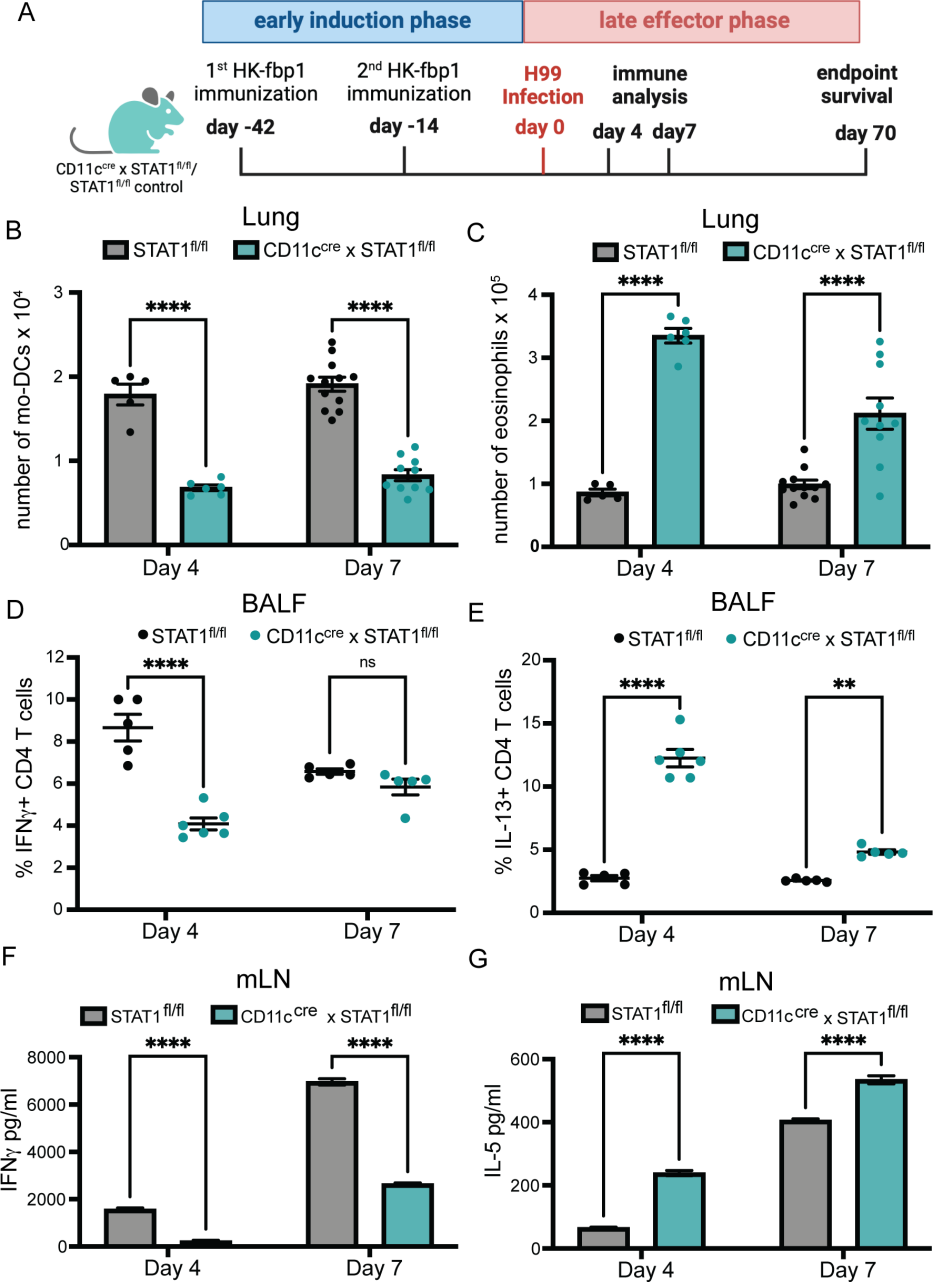
STAT1 expression on CD11c^+^ cells is important for maintaining Th1 responses. (**A**) Graphical summary of the experimental approach. CD11c^cre^ × STAT1^fl/fl^ and STAT1^fl/fl^ control mice were vaccinated with HK-fbp1 on day −42 and a boost vaccine on day −14. On day 0, mice were infected with live 10^4^ H99. Mice were sacrificed on day 4 and day 7 post-H99 infection. (B to C) The total number of Mo-DCs (**B**) and eosinophils (**C**) were examined by flow cytometry on days 4 and 7. Each symbol represents one mouse. (D to E) Cytokine expression in CD11c^cre^ × STAT1^fl/fl^ and STAT1^fl/fl^ control mice was analyzed by ICCS and measured by flow cytometry at each time point. The frequencies of IFNγ- and IL-13-producing CD4^+^ T cells in BALF are shown. Each symbol represents one mouse. (F to G) CD4^+^ T cells were purified from lung-draining lymph nodes from CD11c^cre^ × STAT1^fl/fl^ or STAT1^fl/fl^ control mice at each time point and stimulated with *Cryptococcus* antigens for 72 hours. IFN-γ and IL-5 levels were measured in culture supernatant by ELISA. The data shown are cumulative from two independent experiments with five mice per group and are depicted as the mean values ± standard errors of the means. ns, not significant; **, *P* < 0.01; ****, *P* < 0.0001 (determined by two-way ANOVA nonparametric test for multiple comparisons using Prism software).

Thus far our data suggest that CD11c^+^ cells, which include CCR2^+^ Mo-derived cells, are key for the activation of T cells in response to HK-fbp1 immunization via roles during initial priming and re-expansion after infection. Our transcriptomic analysis indicated that STAT1-dependent signals downstream of IFNγ activation are significantly induced in TD-AM and Μo-AM after HK-fbp1 immunization. Furthermore, previous studies indicate that CD11c^+^ cells are important effectors of fungal cell control where IFNγ-educated, M1 macrophages are considered key mediators of phagocytosis and killing of *Cryptococcus* yeast cells (51–54). Therefore, we hypothesized that CD11c^+^ cells are key responders of IFNγ/STAT1-induced signals for the orchestration of HK-fbp1 vaccine-induced protection. To start addressing this hypothesis, we tested the impact of selected STAT1 deficiency on CD11c^+^ cells on the activation of HK-fbp1-induced protection. We focused on examining how impaired STAT1 expression on CD11c^+^ cells affected innate cell differentiation, T cell re-expansion, and fungal dissemination in vaccinated mice at day 4 and day 7 post-H99 infection. We vaccinated CD11c^cre^ × STAT1^fl/fl^ and STAT1^fl/fl^ control mice with HK-fbp1 on day −42 and a boost vaccine on day −14. On day 0, these mice were infected with live H99. Mice were sacrificed on day 4 and day 7 post-H99 infection (p.i.) for various immune analyses (Fig. 6A). We found that removal of STAT1 signaling on CD11c^+^ cells affected monocyte recruitment and total number of AMs in the lung on day 7 p.i. (Fig. S6A and B) but showed no significant changes at day 4. More importantly, removal of STAT1 signaling on CD11c^+^ cells affected monocyte differentiation into Mo-DCs in the lungs, which started at day 4 p.i. and continued until day 7 p.i. (Fig. 6B). We also found increased numbers of eosinophils in the lungs of CD11c^cre^ × STAT1^fl/fl^ mice compared to their STAT1^fl/fl^ littermate controls (Fig. 6C). In addition to changes in Mo-DCs and eosinophils, we observed that CD11c^cre^ × STAT1^fl/fl^ mice had significant reductions in the frequency of IFNγ-producing CD4^+^ T cells (Fig. 6D), reductions in the frequency of IFNγ-producing CD8^+^ T cells (Fig. S6D), and an increase of IL-13-producing CD4^+^ T cells in the BALF (Fig. 6E). However, IL-17A-producing CD4^+^ T cells remained comparable in CD11c^cre^ × STAT1^fl/fl^ mice compared to the vaccinated STAT1^fl/fl^ control mice (Fig. S6C), indicating that Th17 responses were not affected. We also examined recall and *Cryptococcus*-specific T-cell responses in the mLNs. Consistent with findings in BALF, we found decreased Th1 (Fig. 6F) and increased Th2 responses (Fig. 6G) in lymph nodes from CD11c^cre^ × STAT1^fl/fl^ mice as compared to their STAT1^fl/fl^ controls. These data indicate that STAT1 responsiveness in CD11c^+^ cells is required for their effector function during the effector phase of vaccine-induced production. We thus set out to examine whether STAT1 signals on CD11c^+^ cells were also required for the activation of T cell responses after the initial immunization with HK-fbp1. Surprisingly, we found that the removal of STAT1 responsiveness on CD11c^+^ cells did not change the cytokine profile of CD4^+^ T cells in the BALF and mLNs at day 7 post-Hk-fbp1 vaccination. We observed that CD4^+^ and CD8^+^ T cell priming, differentiation, and recruitment to the lung was equivalent in CD11c^cre^ × STAT1^fl/fl^ as compared to control STAT1^fl/fl^ mice during the early induction phase (Fig. S6E through L). These results suggest that the initial differentiation of CD4^+^ T cells was not affected by the abolished STAT1 responsiveness on CD11c^+^ cells. Therefore, the removal of STAT1 signaling on CD11c^+^ cells did not affect the protective host response in the early induction phase while it did have significant impacts on T cell responses after infection.

**Fig 7:**
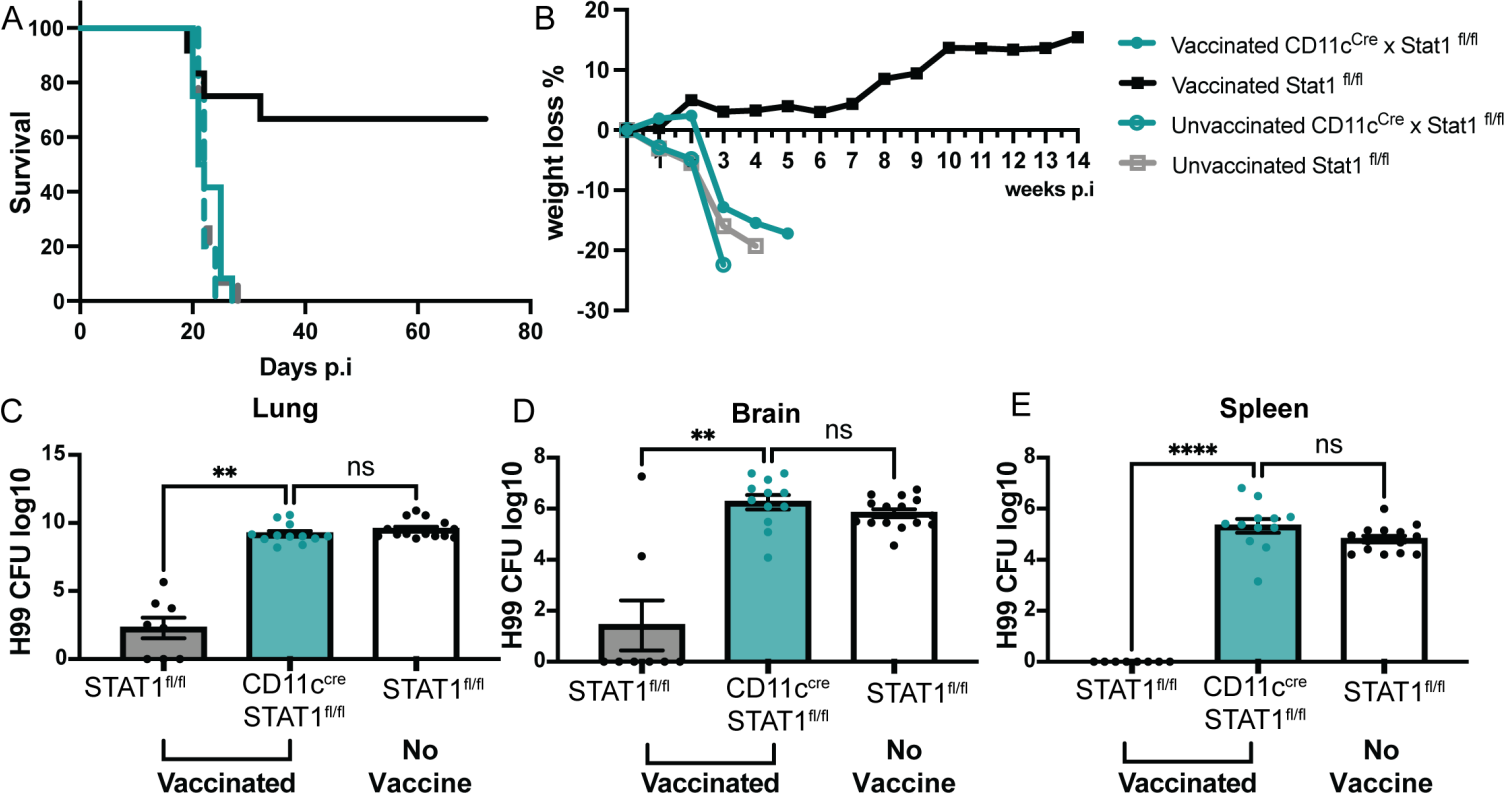
STAT1 expression on CD11c^+^ cells is critical for controlling fungal dissemination and overall survival. CD11c^cre^ × STAT1^fl/fl^ and STAT1^fl/fl^ control mice were vaccinated with HK-fbp1 on day −42 and a boost vaccine on day −14. On day 0, mice were infected with live 10^4^ *C. neoformans* H99. (**A**) Survival curve of vaccinated CD11c^cre^ × STAT1^fl/fl^ (solid, teal line), vaccinated STAT1^fl/fl^ (solid black line), unvaccinated CD11c^cre^ × STAT1^fl/fl^ (dashed teal line), and unvaccinated STAT1^fl/fl^ (dashed gray line) after challenge with H99. (**B**) Changes in mouse body weight over time after H99 infection. Data for vaccinated and unvaccinated CD11c^cre^ × STAT1^fl/fl^ and STAT1^fl/fl^ is shown and represented as defined in the figure legend. (C to E) H99 fungal burden was examined in lungs (**C**), brain (**D**), and spleen (**E**) at day 7 post-H99 infection. Each symbol represents one mouse. The data shown are cumulative from two independent experiments with five mice per group and are depicted as the mean values ± standard errors of the means. **, *P* < 0.01****; *P* < 0.0001 (C to E, as determined by one-way ANOVA nonparametric test for multiple comparisons using Prism software).

In light of these observations, we set out to determine the overall impact of STAT1 deficiency on CD11c^+^ cells to vaccine-induced protection. Strikingly, HK-fbp1 immunization was unable to induce any protection in mice deficient in STAT 1 expression in CD11c^+^ cells in contrast to the significant protection induced in control STAT1^fl/fl^ mice (Fig. 7A). We tracked the changes in animal body weight weekly throughout the experiment. These weight changes reflected overall animal health status and were correlated with the survival curve (Fig. 7B). Fungal dissemination in mice is associated with overall survival to cryptococcosis. We compared fungal burden at the site of infection (lung), systemic infection (spleen), and fungal meningitis (brain) in CD11c^cre^ × STAT1^fl/fl^ mice and their STAT1^fl/fl^ controls. We found that vaccinated, STAT1^fl/fl^ controls were able to prevent fungal dissemination from the lung (Fig. 7C) and did not show fungal cell spreading to the spleen and brain. Meanwhile, mice that lack STAT1 signaling in CD11c^+^ cells were unable to control disseminated infection (Fig. 7D and E). Altogether, our observations indicate that STAT1 responsiveness by CD11c^+^ cells is crucial for HK-fbp1 vaccine-induced protection against virulent challenge with H99 via effects that include (i) promoting protective innate responses characterized by higher Mo-DCs and low eosinophilia, (ii) protective Th1 expansion in the MLN and infiltration to the airways, and (iii) control of fungal dissemination from the lung.

In aggregate, based on all the results we propose a model to explain the roles that IFNγ and innate cells play in homologous protection elicited by the HK-fbp1 vaccine. During the early induction phase of HK-fbp1 vaccination, neutrophils and monocytes serve as important sources of early IFNγ production. Early IFNγ expression was essential for Mo-DC expansion and to initiate a primary, protective Th1 response. After mice were challenged with H99, during the late effector phase, the data demonstrated the crucial contribution of CD11c^+^ innate cells and the importance of STAT1 responsiveness on these cells for HK-fbp1 vaccine-induced protection. Our data indicate that STAT1 signaling on CD11c^+^ cells which includes Mo-AM and TD-AMs, is important for maintaining the balance of protective T cell responses during re-expansion after challenge with H99, and for controlling fungal dissemination to extrapulmonary sites.

## DISCUSSION

In this study, we uncovered novel and essential functions for neutrophils, CCR2^+^ inflammatory monocytes, their derivative Mo-DCs, and CD11c^+^ innate cells in a model of HK-fbp1 vaccine-induced protection against *C. neoformans* infection. Cellular sources of IFNγ are very well established in other models, and it is thought to be produced primarily by lymphocytes like NK cells, CD4^+^ T cells, and CD8^+^ T cells. Myeloid cells are usually viewed as targets rather than producers of IFNγ. However, some studies observed the production of IFNγ by macrophages (55, 56). For example, mouse resident peritoneal macrophages and alveolar macrophages can also produce IFNγ upon LPS stimulation (57). In contrast to macrophages, the significance of granulocytes as a potential source of IFNγ has been underestimated. *In vitro* studies of human neutrophils stimulated with LPS, TNFα, or IL-12 were found to produce IFNγ (58, 59). A more recent study in a mouse model of toxoplasmosis identified the presence of a nonlymphoid source of IFNγ. By employing flow cytometry and morphological examinations, researchers determined that neutrophils are capable of producing IFNγ in a TLR-independent manner (60). In a model of aspergillosis, our recent studies also revealed that neutrophils can be a source of IFNγ during pulmonary fungal infection (47). In this study, we found that myeloid innate cells such as neutrophils and CCR2^+^ monocytes were able to produce IFNγ at day 3 after mice received HK-fbp1 vaccine. In our model, at day 3 post-HK-fbp1 vaccination, the removal of neutrophils diminished global IFNγ expression in the lung. Removal of CCR2^+^ monocytes also affected IFNγ expression in the lung. Interestingly, the removal of monocytes did not affect neutrophil recruitment but resulted in an impaired capacity for neutrophils to produce IFNγ. This data demonstrated that innate myeloid cells like monocytes and neutrophils can be important sources of IFNγ upon stimulation by a protective fungal vaccine strain. It is possible that the production of IFNγ by neutrophils is regulated by monocytes and their derived cells. Neutrophils are essential to license antifungal monocytes during *Af* infection (61). Depletion of neutrophils during the early stage of *Af* infection decreases monocyte ROS production and type I, II, and III IFN expression levels in the lung (47). Moreover, neutrophil-depleted mice treated with IFNγ had improved CCR2^+^ monocyte antifungal responses, and increased MHC II expression as well as improved control of fungal burden (47). Thus, together with the findings presented in this paper, these observations suggest a conserved response by monocytes and neutrophils where IFNs are key mediators of cellular cross-talk and antifungal immunity not only during infection but also in response to HK-fbp1 vaccination.

IFNγ production is essential for protective Th1 priming against infections. In the context of HK-fbp1-vaccination, early IFNγ expression from innate cells was essential for Mo-DC differentiation, and to initiate a primary, protective Th1 response. In order to discover the mechanism of protective host immunity against challenge with H99 in the HK-fbp1 vaccination mouse model, we used a mouse model in which interferon signaling is conditionally knocked out on specific innate cell populations (CD11c^cre^ × STAT1^fl/fl^ mice), and carefully examined host immune responses. In the late effector phase (after mice received HK-fbp1, and were challenged with H99), our data demonstrated the crucial contribution of CD11c^+^ innate cells and their requirement for STAT1 in order to activate protective antifungal immunity. These protective responses include enhanced Mo-DC differentiation and re-expansion of protective Th1 responses, therefore, controlling fungal dissemination. Given the key role of IFNγ in HK-fbp1 vaccine-induced protection, we hypothesize that the essential role of STAT1 on CD11c^+^ cells seen in this study is likely due to IFNγ-dependent engagement of STAT1. However, STAT1 is also key for type I and III IFN responses and a potential role for these cytokines cannot be ruled out by the approaches used in this study. In addition, other cytokines, including IL-6, IL-10, and IL-21, can also engage STAT1 and can similarly not be fully excluded from shaping the responses measured in this study. An additional point of consideration is that STAT1 signals can regulate diverse antifungal responses by phagocytes that go beyond direct cytokine responsiveness (52–54).

Our work extends the knowledge of the importance of IFN signaling in innate cells in a vaccine-induced protection mouse model. During the early induction phase, innate components like monocytes and neutrophils play important roles in initiating a protective Th1 immune response. During the late effector phase, CD11c^+^ cells act as responders for IFNs and are essential for the secondary protective re-expansion of T cells. Intriguingly, we observed that monocytes give rise to Μo-AM that show a persistent IFN signature gene expression at day 30 post-immunization. TD-AMs also showed IFN responsiveness and it is likely that the impact of depletion of CD11c^+^ seen in CD11c-DTR mice is at least in part due to the removal of these important alveolar macrophage populations. An additional consideration for the data presented in this paper is that the CD11c^+^ marker is expressed on not only macrophages and DCs, but also on T cells (iNKT cells, and γδ T cells) and subsets of B cells. So far, the functions of these cell subsets during fungal infection are poorly understood. Thus, further studies will be needed to investigate the potential contributions of these different CD11c^+^ populations.

Our aggregate findings uncovered important roles for neutrophils, monocytes, and monocyte-derived cells as both sources and targets of the protective effects of IFNγ in response to HK-fbp1 vaccination. This new insight will help guide future studies and assessments on the utility of HK-fbp1 as an antifungal vaccine for susceptible patients.

## MATERIALS AND METHODS

### CCR2^+^ inflammatory monocyte and CD11c^+^ cell depletion

The CCR2 depleter (CCR2-DTR) and CCR2 reporter (CCR2-GFP) strains were generated on the C57BL/6 (WTB6) background as previously described (48). Control animals for CCR2-DTR experiments were sex and age-matched, nontransgenic littermates. CD11c-DTR mice in C57BL/6 background were from Dr. Kumamoto’s Laboratory at Rutgers University. CD11c-DTR mice were generated with a targeting construct containing a floxed DTR-IRES-GFP cassette containing a splice acceptor that was knocked into the first intron of the CD11c gene, known as integrin (*Itgax*) (62). For depletion of CCR2^+^ or CD11c^+^ cells, CCR2-DTR mice and control CCR2-DTR negative littermates; or CD11c-DTR mice and their littermate controls, received 250 ng of diphtheria toxin i.p. 1 day prior to infection and every other day thereafter in order to maintain depletion. Diphtheria Toxin was purchased from List Biological Laboratories (Campbell, CA), and reconstituted at 1 mg/mL in PBS.

### Neutrophil depletion

WTB6 background mice were purchased from Jackson Laboratories. For 3 days of depletion, WTB6 mice were i.p. injected with 100μg 1A8 antibody on day −1 and day +1. On day 0, mice were vaccinated with HK-fbp1. On day 0 and day +2, mice were i.p. injected with 100μg of MAR18.5 antibody. Control antibody 2A3 100μg was i.p. injected into mice on day −1 and day +1 (27). All antibodies were purchased from BioXcell.

### IFNγ neutralization

WTB6 background mice were purchased from Jackson Laboratories. Mice were vaccinated with HK-fbp1 on day 0 and received 200μg of anti-IFNγ (XMG1.2) or isotype control antibody (rat IgG1) for neutralization of IFNγ on days +1, +2, +3, and +5. Control mice were treated with isotype control antibodies using the same dose and regimen as done for anti-IFNγ neutralization. All antibodies were purchased from BioXcell. The dose and timing of XMG1.2 used were in accordance with a previously published study where effective IFN-γ neutralization was achieved as demonstrated by comparable mortality to *T. gondii* infection in IFNγ^−/−^ and α-IFNγ-neutralized mice (45).

### Monocyte fate mapper mice and RNA-seq

CX3CR1^creER^ × R26 TdTomato or CCR2^CreER^ × R26 TdTomato (monocyte fate mappers) were injected with 2.5 mg Tamoxifen (Sigma, T5648) per animal in 100 ml corn oil (Sigma) on day −1 before vaccination. On day 0, mice were vaccinated intranasally with 5 × 10^7^ HK-fbp1. At various times after vaccination cells in lung tissue were identified as Mo-AM (live CD45^+^ SiglecF^+^ CD11c^+^ CD64^+^ F4/80^+^ NK1.1^−^ TdT^+^), TD-AM (live CD45^+^ SiglecF^+^ CD11c^+^ CD64^+^ F4/80^+^ NK1.1^−^ TdT^−^), Mo-DC (live CD45^+^ SiglecF^−^ CD11c^−^ NK1.1^−^ TdT^+^), and non-monocyte derived DCs (live CD45^+^ SiglecF^−^ CD11c^−^ NK1.1^−^ TdT^−^). On day 30, mice were sacrificed, and cells were isolated from lung tissue for transcriptional analysis. Mo-AM and TD-AM were isolated >98% purity using a BD FACSAria II cell sorter. RNA processing for library generation and sequencing on an Illumina HiSeq instrument was done by the Genomic Research Core facility as described (61, 63). Pathway analysis of gene expression profiles was done using Ingenuity Pathway Analysis software (Qiagen). For each group, three different biological replicates were examined, and differentially regulated gene clusters were identified.

### Immune cell population identification

Immune cell populations in lung cell suspensions were identified as live CD45^+^ DAPI^−^ cells: monocytes (CD11b^+^ CCR2^+^ Ly6C^+^ Ly6G^−^); Mo-DCs (CD11b^+^ CCR2^+^ Ly6C^+^ Ly6G^−^ CD11c^+^ MHC class II^+^); AM (CD11c^+^ SiglecF^+^); eosinophils (CD11c^−^ SiglecF^+^); CD4^+^ T cells (CD11b^−^ Thy1.2^+^ CD4^+^ CD8^−^); CD8^+^ T cells (CD11b^−^ Thy1.2^+^ CD4^−^ CD8^+^); FATE MAPPER: monocyte-derived AM (CD11c^+^ SiglecF^+^ Td-Tomato^+^), tissue derived AM (CD11c^+^ SiglecF^+^ Td-Tomato^−^). BALF cell populations were identified as CD4^+^ T cells (Thy1.2^+^ CD4^+^ CD8^−^); CD8^+^ T cells (Thy1.2^+^ CD4^−^ CD8^+^).

### Analysis of cytokines and RNA expression

Total RNA from the lungs was extracted with Trizol (Invitrogen). Relative mRNA levels were determined by quantitative reverse-transcription polymerase chain reaction (qRT-PCR). One microgram of total RNA was reverse transcribed using a High Capacity cDNA Reverse Transcription Kit (Applied Biosystems). Taq Man Fast Universal PCR Master Mix (2×) No Amp and TaqMan probes (Applied Biosystems) for each gene were used and normalized to GAPDH. Gene expression was calculated using the ΔΔCT method relative to gene expression in uninfected controls. For cytokine measurements in lung tissue, we performed ELISAs on lung homogenates according to the manufacturer’s instructions.

### HK-fbp1 vaccination preparation and live H99 infection

The *C. neoformans* fbp1 mutant strain was heat-killed following a previously described procedure (34, 44). Fungal cells from yeast extract-peptone-dextrose (YPD) overnight cultures were precipitated and washed twice with sterile PBS. The cell suspension at 1 × 10^9^ /mL concentration was heated on a hot plate at 75°C for 90 minutes. For survival experiments, mice were vaccinated intranasally with 5 × 10^7^/50 µL heat-killed fungal cells (HK-fbp1) at day −42 unless otherwise specified. Each group of mice was vaccinated again with the same dose of HK-fbp1 at day −14. To prepare cryptococcal fungal cells for infection *C. neoformans* H99 were cultured in YPD overnight and washed twice with sterile PBS. On day 0, mice were challenged with 10^4^/50 µL live H99 cells intranasally. Infected mice were weighed and monitored daily for disease progression.

### Fungal burden (CFU)

Infected tissues were isolated and homogenized in 3 mL of PBS for 1 minute. The tissue suspensions were serially diluted and plated onto YPD agar medium with ampicillin and chloramphenicol, and colonies were counted after 3 days of incubation at 30°C.

### Lung cell isolation and flow cytometry

Lung samples were minced in PBS with 3 mg/mL of collagenase type IV (Worthington) and were incubated at 37°C for 45 minutes to obtain single-cell suspensions. After digestion, lung suspensions were lysed of red blood cells. All antibodies were purchased from BD Biosciences. The staining protocols included combinations of the following antibodies: Gr-1 (RB6-8C5), Ly6C (AL-21), Ly6G (1A8), CD11b (M1/70), CD11c (N418), CD45 (30-F11), MHC Class II I-A/I-E (M5/11.415.2), NK1-1 (PK136), Siglec F (E50-2440), F4/80 (BM8; BioLegend), Thy1.2 (53-2.1), CD4 (RM4-4), CD8a (53-6.7). DAPI (Life Technologies) was used as a viability control. Samples were collected on a BD LSRFortessa X-20 and analyzed using FlowJo software.

### Intracellular cytokine staining of T cells harvested in BALF

BALF was collected in 5 mL of PBS buffer using a catheter inserted into the trachea of the animal post-euthanasia, and airway-infiltrating cells were lavaged with 1 mL of 1× PBS at a time for a total volume of 5 mL. RBCs were removed using RBC lysis buffer. BALF cells were then plated in a 96-well round-bottom tissue culture plate and restimulated using a BD leukocyte activation cocktail containing BD GolgiPlug (BD Biosciences) according to the manufacturer’s instructions. Six hours after activation, BALF cells were surface stained with fluorescently labeled antibodies against Th1.2 (53-2.1), CD4 (RM4-4), and CD8α (53-6.7). Samples were fixed in 1% paraformaldehyde overnight. Prior to intracellular staining, the samples were permeabilized with 1× BD Perm/Wash buffer according to the manufacturer’s instructions. Intracellular cytokine staining (ICCS) was done using fluorescently labeled antibodies against IFNγ, IL-17A, and IL-13 diluted in 1× BD Perm/Wash buffer for 45 minutes on ice.

### CD4^+^ T cell isolation and recall response

Antigen-presenting cells were prepared from the spleens of syngeneic, uninfected donor mice. Splenic cell suspensions were depleted of T cells by antibody complement-mediated lysis. Splenic cells were incubated with anti-Thy1.2 antibody and rabbit complement (Low Tox; Cedarlane Labs) at 37°C for 1 hour. CD4^+^ T cells in mLN were extracted using a negative-sorting CD4^+^ isolation kit (Miltenyi Biotec, Inc.). CD4^+^ T cell isolation was done following the manufacturer’s instructions. Purified CD4^+^ T cells (2 × 10^5^) were cultured with T cell-depleted antigen-presenting cells (3 × 10^5^) in RPMI medium containing 10% fetal calf serum (FCS), penicillin-streptomycin (2,200 U/mL, Gibco), and gentamicin sulfate solution (1 mg/mL). The cultures were plated in flat-bottom 96-well plates and incubated at 37°C with 5% CO_2_ for 72 hours. To measure cryptococcal-specific CD4^+^ T cell responses, CD4^+^ antigen-presenting cell cultures were incubated with sonicated (Qsonica sonicator Q55) H99 yeast as a source of fungal antigens. The amount of antigen used was adjusted to a multiplicity of infection of 1:1.5 (antigen-presenting cell/yeast ratio). The fungal growth inhibitor voriconazole was used at a final concentration of 0.5 mg/mL to prevent any fungal cell overgrowth during the culture period. After 72 hours of culture at 37°C with 5% CO_2_, supernatants were collected for cytokine analysis by enzyme-linked immunosorbent assay (ELISA (IL-2 and IL-5, BD OptEIA; IL-17A and IFNγ, ThermoFisher) following the manufacturer’s instructions.

### Statistics

Survival data from the murine experiments were statistically analyzed between paired groups using the log-rank (Mantel-Cox) test with PRISM version 8.0 (GraphPad Software) (*P* values of 0.05 were considered statistically significant). Statistical analysis of *in vivo* and *in vitro* parameters of antifungal immunity was performed using a nonparametric Mann-Whitney test in GraphPad Prism version 8.0 software.

## Data Availability

RNA-seq data hasve been deposited as GEO project GSE271485.

## SUPPLEMENTAL MATERIAL

Supplemental figures mbio.01944-24-s0001.pdf Figures S1 to S6.

ASM does not own the copyrights to Supplemental Material that may be linked to, or accessed through, an article. The authors have granted ASM a non-exclusive, world-wide license to publish the Supplemental Material files. Please contact the corresponding author directly for reuse.
