# Supplementary material for: Innate cells and STAT1-dependent signals orchestrate vaccine-induced protection against invasive Cryptococcus infection
Source: mBio. 2024 Sep 26;15(10):e01944-24. doi: 10.1128/mbio.01944-24 (PMC11481872; doi:10.1128/mbio.01944-24)
Supplement: Supplemental figures — Figures S1 to S6. [file mbio.01944-24-s0001.pdf]

Wang et al Supplementary Figure

Fig 1S

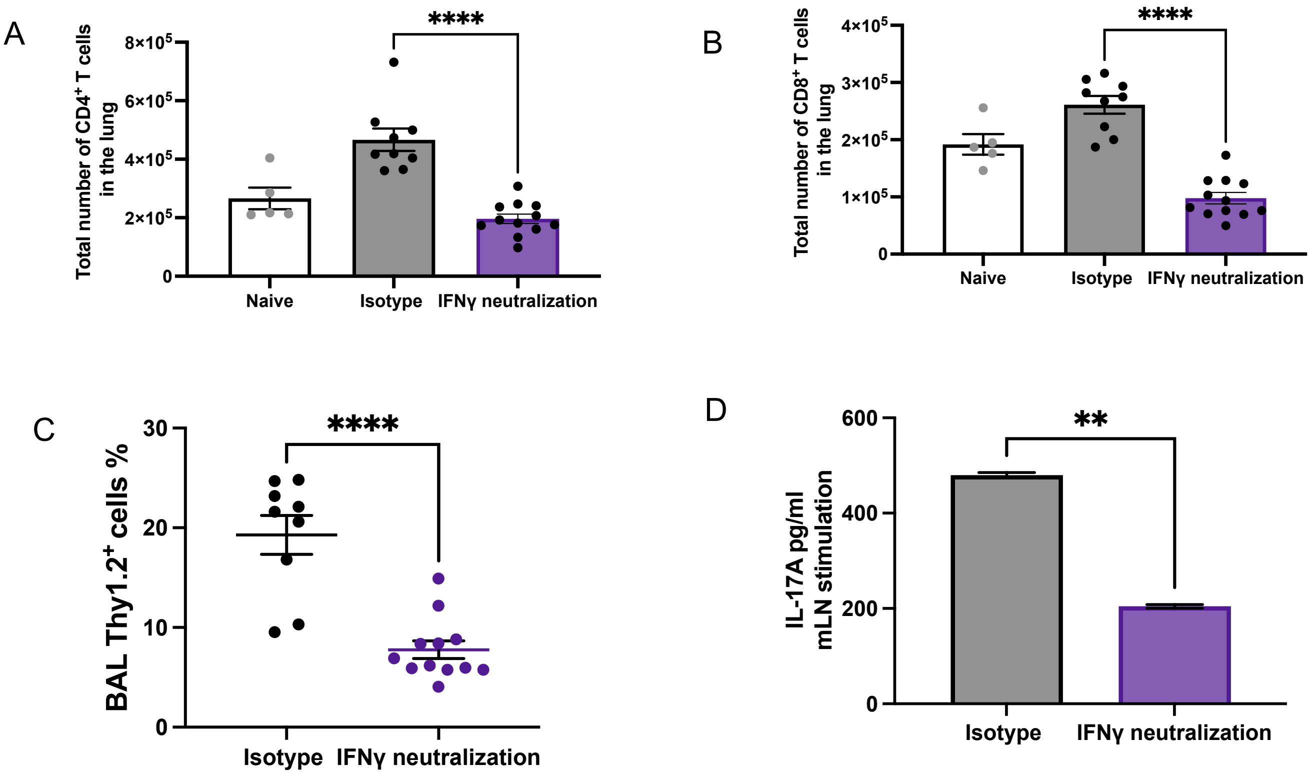

Figure S1. WT B6 Mice were vaccinated with HK-fbp1 and sacrificed on day 7 post vaccination. Mice received 200 ug of XMG1.2 or isotype control antibody for neutralization of IFN-γ on day +1, +2, +3, and +5. (A to B) Total number of $CD4^+$ T cells and $CD8^+$ T cells recovered from the lung at day3 and day7 post infection as measured by flow cytometry. Immune cell populations were identified as described in the Material and Methods section. (C) Plot shows the percentage of $Thy1.2^+$ T cells recovered in Bronchoalveolar lavage fluid (BALF) as examined by flow cytometry. (D). $CD4^+$ T cells were purified from lung draining lymph nodes and stimulated with *Cryptococcus* antigens for 72 hours as detailed in the materials and methods section. Secreted IL-17A levels were measured in culture supernatant by ELISA. The data shown are cumulative for two independent experiments with five mice per group and are depicted as the mean values ± standard errors of the means. **, $P < 0.01$, ****, $P < 0.0001$ (determined by nonparametric t-test using Prism software).

Supplementary Fig 2

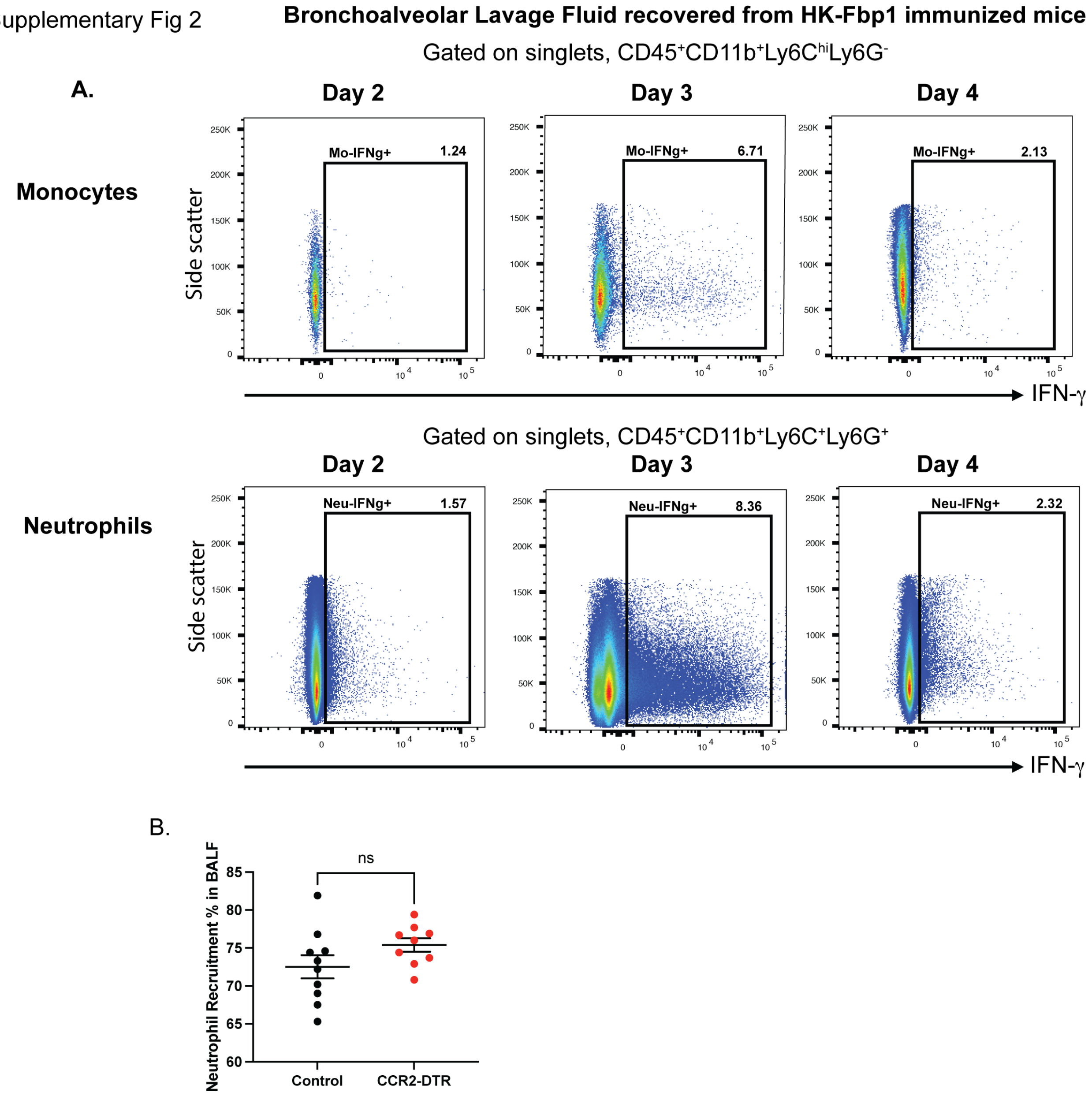

Figure S2. (A). WT B6 mice were vaccinated with HK-fbp1 5x10^{7} at day 0, and sacrificed on day 2, day 3, day 4, and day 5 post vaccination. Representative FACS plots of IFNγ-producing monocyte and IFNγ-producing neutrophils in the BALF on each time points. (B) Percentage of neutrophils in the BALF. ns, not significant as determined by nonparametric t-test using Prism software

S3

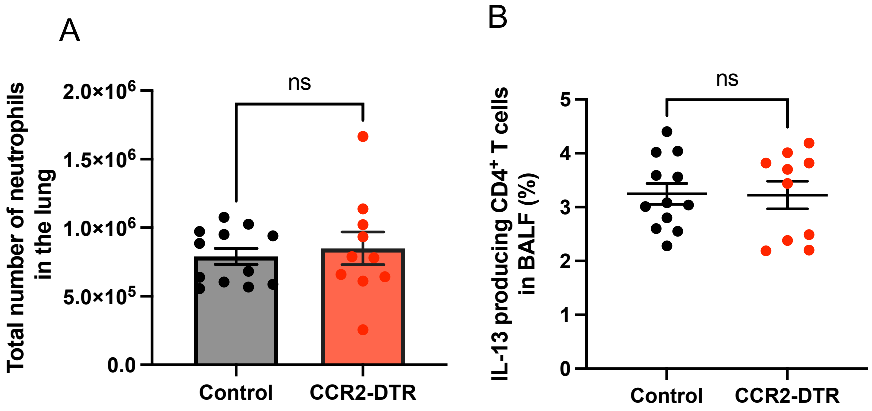

Figure S3. WTB6 mice were vaccinated with HK-fbp1 5x10^{7} on day0 and sacrificed on day 7 post vaccination. CCR2-DTR mice and control CCR2-DTR negative littermates received 250 ng of diphtheria toxin i.p. day -1 prior to vaccination and every other day after vaccination to maintain depletion. (A) Total numbers of neutrophils in the lungs at day7 post infection measured by flow cytometry. (B). Percentage of IL-13-producing CD4^{+} T cells in CCR2-DTR mice and littermate controls were analyzed by ICCS and measure by flowcytometry. Each symbol represents one mouse, data is cumulative for two independent experiments. ns, not significant. (determined by nonparametric t-test for comparison)

S4

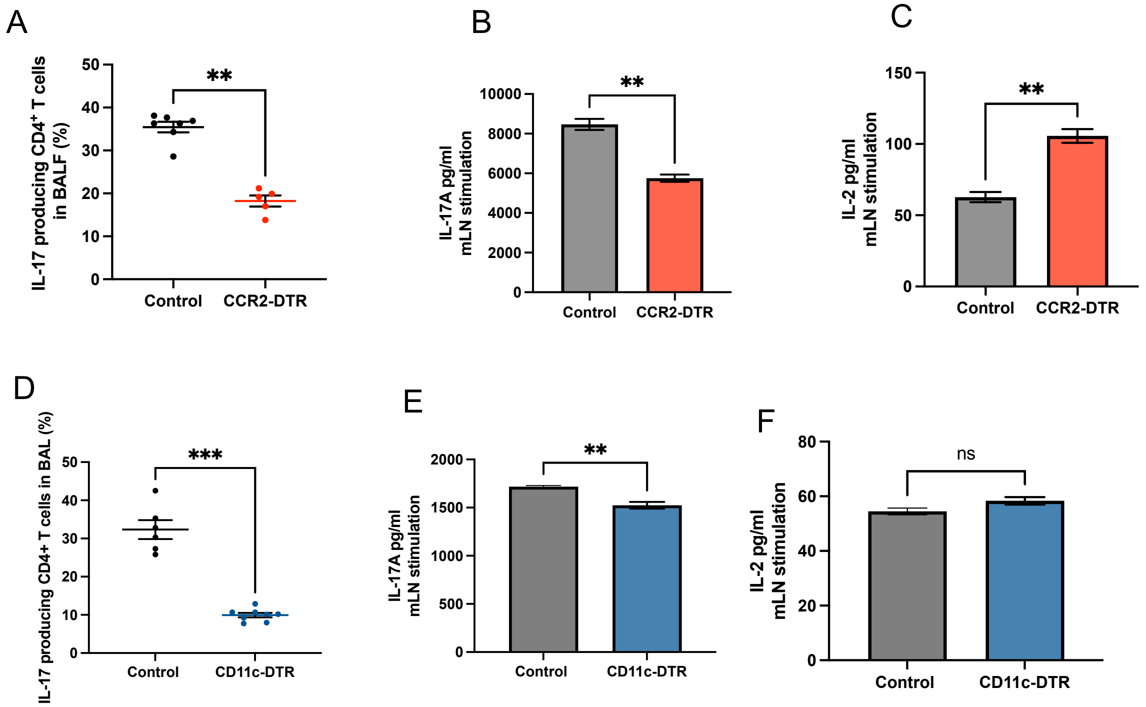

Figure S4. (A). CCR2-DTR mice and control CCR2-DTR negative littermates were vaccinated with 5 x 10^7 HK-fbp1 on day -42 and boosted with HK-fbp1on day -14. On day 0, mice were infected with $10^{4,}$ live, H99. All mice received 250 ng of diphtheria toxin i.p. on day -1, +1, +3 and sacrificed on day 4 post H99 infection. (A) Graph shows percentage of IL-17A-producing $CD4^+$ T cells in airways as analyzed by ICCS and flowcytometry. Each symbol represents one mouse. (B and C). $CD4^+$ T cells were isolated from lung-draining lymph nodes of CCR2-DTR and littermate controls and re-stimulated ex vivo with Cryptococcus antigens. IL-17A and IL-2 levels were examined in culture supernatant by ELISA 72 hrs after culture initiation. Data is cumulative for two independent experiments and expresses as mean±SD. (D). CD11c-DTR and their WT littermate control mice were vaccinated with HK-fbp1 on day -42 and boosted with HK-fbp1 vaccine on day -14. On day 0, mice were infected with 10^4 , live H99. All mice received 250 ng of diphtheria toxin i.p. on day -1, +1, +3 and were sacrificed on day 7 post H99 infection. Data shows percentage of IL-17A-producing $CD4^+$ T cells in airways as analyzed by ICCS and measured by flowcytometry. Each symbol represents one mouse (E to F). *Cryptococcus*-specific $CD4^+$ T cell recall responses were measured in T cells isolated from lung-draining lymph nodes. IL-17A and IL-2 levels were examined in culture supernatant by ELISA. Data shown is cumulative for two independent experiments with five mice per group. ns, not significant, **, P < 0.01, ****, P < 0.0001; (determined by nonparametric t-test using Prism software).

S5

Supplementary Fig 5

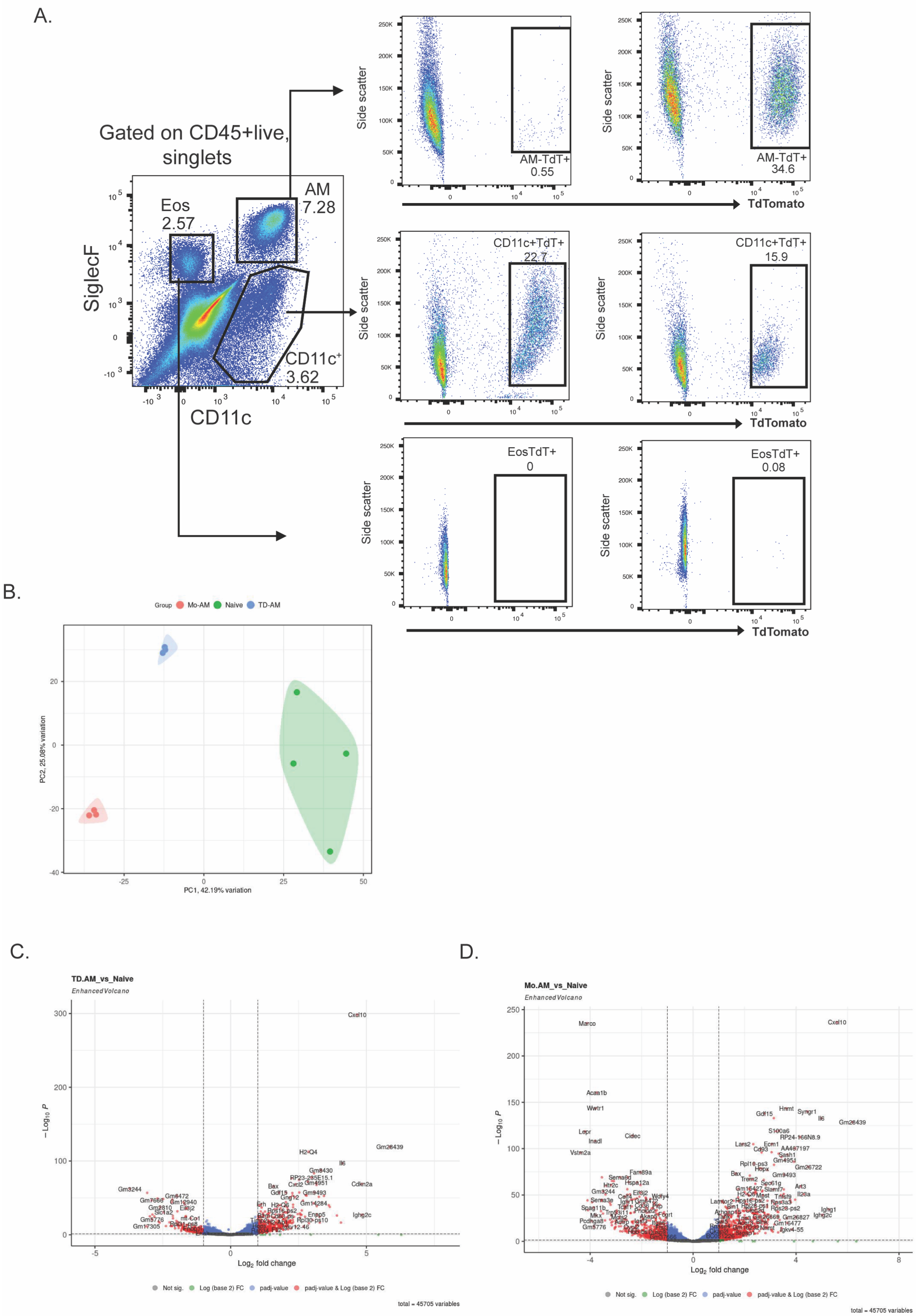

Figure S5. CCR2^{creER} x R26TdTomato$^{fl/fl}$ mice were vaccinated with HK-fbp1 and sacrificed on day 30 post vaccination. Representative FACS plots of gating strategy of eosinophils (CD45^{+} DAPI^{-} CD11c^{-} SiglecF^{hi}), AMs (CD45^{+} DAPI^{-} CD11c^{+} SiglecF^{hi}), and CD11c^{+} cells (CD45^{+} DAPI^{-} CD11c^{+} SiglecF^{lo}) in the lung at day 30 post vaccination and naïve control mice. B. Principal component analysis of global gene expression in each sample. Each symbol is for data from one independent biological replicate per condition. C and D. Volcano plot of differentially expressed genes between Naïve Vs TD-AM, and Naïve Vs Mo-AM isolated from lung of HK-fbp1 vaccinated mice.

S6

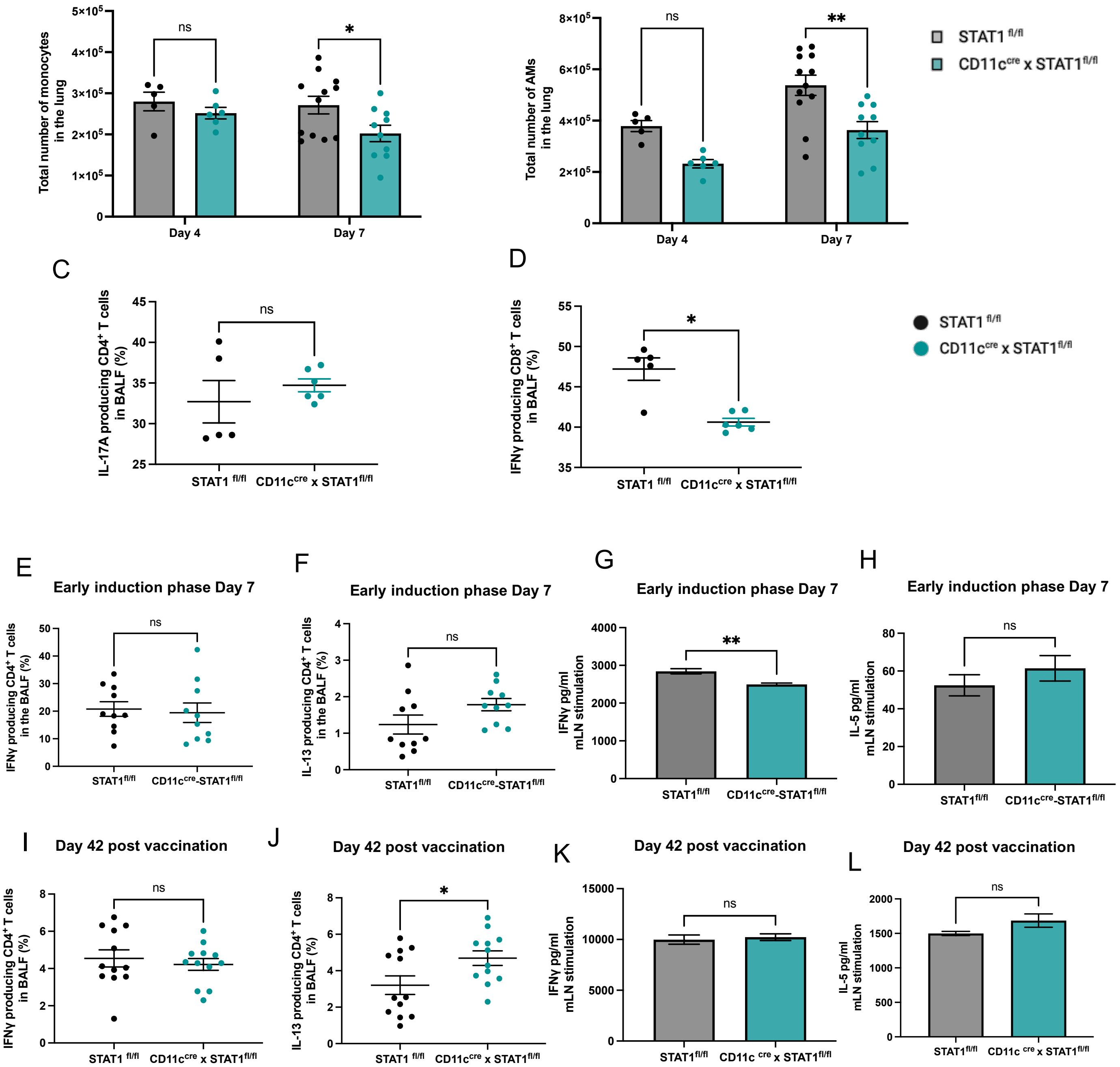

Figure S6. (A to D). CD11c^{cre} x STAT1$^{fl/fl}$ and STAT1$^{fl/fl}$ control mice were vaccinated with HK-fbp1 on day -42 and a boosted with HK-fbp1 vaccine on day -14. On day 0, mice were infected with live 10^4 H99. Mice were sacrificed on day 4 and day 7 post H99 infection. (A to B) Total number of monocytes and AMs in the lung were examined by flow cytometry. (C to D) Cytokine expression in CD11c^{cre} x STAT1$^{fl/fl}$ and STAT1$^{fl/fl}$ control mice was analyzed by ICCS. The frequencies of IL-17A- producing CD4$^+$ T and IFNγ-producing CD8$^+$ T cells in BALF were analyzed as shown. Each symbol represents one mouse. (E to H) CD11c^{cre} x STAT1$^{fl/fl}$ and STAT1$^{fl/fl}$ control mice were vaccinated with HK-fbp1. Mice were sacrificed on day 7 post HK-fbp1 vaccination. (E to F). The frequencies of IL-17A- producing CD4$^+$ T and IL-13-producing CD4$^+$ T cells in BALF at day 7 post vaccination were analyzed as shown. Each symbol represents one mouse (G to H). CD4$^+$ T cells were purified from lung draining lymph nodes in CD11c^{cre} x STAT1$^{fl/fl}$ or STAT1$^{fl/fl}$ control mice at day 7 post vaccination and stimulated with *Cryptococcus* antigens for 72 hours. IFN-γ level and IL-5 level were measured in culture supernatant by ELISA. Data shown is cumulative for two independent experiments and is expressed as mean ± SD. (I to L) CD11c^{cre} x STAT1$^{fl/fl}$ and STAT1$^{fl/fl}$ control mice were vaccinated and boosted with HK-fbp1 vaccine. Mice were sacrificed on day 42 post initial HK-fbp1 vaccination to examine the maintenance of T cell responses after vaccination. (I to J) The frequencies of IL-17A- producing CD4$^+$ T and IL-13-producing CD4$^+$ T cells was examined in BALF at day 42 post vaccination by ICCS and flowcytometry. Each symbol represents one mouse. (K to L). CD4$^+$ T cells were purified from lung draining lymph nodes isolated from CD11c^{cre} x STAT1$^{fl/fl}$ or STAT1$^{fl/fl}$ control mice at day 32 post vaccination and stimulated with *Cryptococcus* antigens for 72 hours. IFN-γ level and IL-5 level were measured in culture supernatant by ELISA. Data is cumulative of two independent experiments and expressed as mean± SD. ns, not significant, *, $P < 0.05$, **, $P < 0.01$, ****, $P < 0.0001$; (A to D. determined by two-way ANOVA analysis of nonparametric test for multiple comparisons; E to L determined by nonparametric t-test for comparison using Prism software
